# Batch effect detection and correction in RNA-seq data using machine-learning-based automated assessment of quality

**DOI:** 10.1186/s12859-022-04775-y

**Published:** 2022-07-14

**Authors:** Maximilian Sprang, Miguel A. Andrade-Navarro, Jean-Fred Fontaine

**Affiliations:** Faculty of Biology, Johannes Gutenberg-Universität Mainz, Biozentrum I, Hans-Dieter-Hüsch-Weg 15, 55128 Mainz, Germany

**Keywords:** Batch effect, Quality control, NGS, RNA-seq, Next-generation sequencing, Machine learning, Bioinformatics, Artifacts, Batch effect origin

## Abstract

**Background:**

The constant evolving and development of next-generation sequencing techniques lead to high throughput data composed of datasets that include a large number of biological samples. Although a large number of samples are usually experimentally processed by batches, scientific publications are often elusive about this information, which can greatly impact the quality of the samples and confound further statistical analyzes. Because dedicated bioinformatics methods developed to detect unwanted sources of variance in the data can wrongly detect real biological signals, such methods could benefit from using a quality-aware approach.

**Results:**

We recently developed statistical guidelines and a machine learning tool to automatically evaluate the quality of a next-generation-sequencing sample. We leveraged this quality assessment to detect and correct batch effects in 12 publicly available RNA-seq datasets with available batch information. We were able to distinguish batches by our quality score and used it to correct for some batch effects in sample clustering. Overall, the correction was evaluated as comparable to or better than the reference method that uses a priori knowledge of the batches (in 10 and 1 datasets of 12, respectively; total = 92%). When coupled to outlier removal, the correction was more often evaluated as better than the reference (comparable or better in 5 and 6 datasets of 12, respectively; total = 92%).

**Conclusions:**

In this work, we show the capabilities of our software to detect batches in public RNA-seq datasets from differences in the predicted quality of their samples. We also use these insights to correct the batch effect and observe the relation of sample quality and batch effect. These observations reinforce our expectation that while batch effects do correlate with differences in quality, batch effects also arise from other artifacts and are more suitably  corrected statistically in well-designed experiments.

**Supplementary Information:**

The online version contains supplementary material available at 10.1186/s12859-022-04775-y.

## Background

Batch effects arise from differences between samples that are not rooted in the experimental design and can have various sources, spanning from different handlers or experiment locations to different batches of reagents and even biological artifacts such as growth location. In the context of sequencing data, two runs at different time points can already show a batch effect [1]. Although these effects can be minimized by good experimental practices and a good experimental design [2], batch effects can still arise regardless and it can be difficult to correct them [3]. Batch effects are known to interfere with downstream statistical analysis, for example by introducing differentially expressed genes between groups that are only detected between batches but have no biological meaning [4, 5]. Vice versa, careless correction of batch effects can result in loss of biological signal contained in the data [6–8]. Proper handling of batched data is thus paramount for successful and reproducible research.

Various methods have been developed to detect or even remove batch effects in genomics data, particularly RNA-seq data and cDNA microarrays. For example, the sva package from Bioconductor [9] can detect and correct effects from several sources of unwanted variation, including batches. It also features functions to correct the data for known batches [10]. Yet, such tools should be used with care because they can mistakenly detect and remove actual biological signals from the data as stated by their authors and as cited above.

Quality control of next-generation sequencing data is an essential but not trivial step in functional genome and epigenome analysis. Many tools have been designed to shed light on the quality of a given sample or file. In previous studies, we used 2642 quality-labeled FASTQ files from the ENCODE project to derive statistical features with different bioinformatics tools well known in the scientific community. We showed that these features have explanatory power over the quality of the data from which they were derived, and built a machine learning classification tool that uses these features as input [11, 12]. With a grid search of multiple machine learning algorithms, from logistic regression to ensemble methods and multilayer perceptrons, we were able to provide a robust prediction of quality in FASTQ files.

As the quality of biological samples could be significantly impacted by batch processing, we hypothesized that an automated machine-learning-based quality classifier could also detect batches in gene-expression datasets and be used to correct the batch effect. Here, we leverage the explanatory power of our machine learning algorithm, to successfully detect batch effects based on quality differences in the samples. In addition, we provide a method for batch correction based on predicted sample quality that we evaluate with 12 published RNA-seq datasets.

## Results

**Fig. 1:**
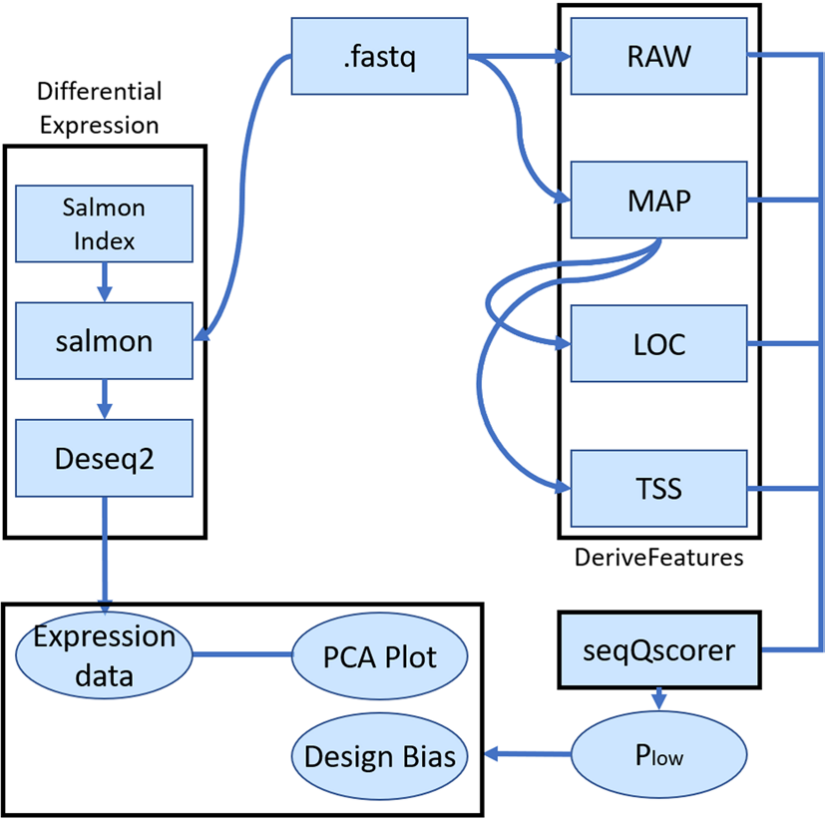
Workflow. Black boxes show components of the overall workflow. DeriveFeatures is a component that uses four bioinformatic tools to derive the four feature sets from the FASTQ files (.fastq): RAW, MAP, LOC, TSS. seqQscorer computes P_low_, the probability of a sample to be of low quality. We used seqQscorer’s generic model, which is derived from 2642 labeled samples and uses a random forest as classification algorithm. We used the salmon tool to quantify gene expression and DESeq2 for rlog normalization [19, 20]

Our workflow to process the data and derive low-quality scores P_low_ by biological sample is depicted in Fig. 1. P_low_ is a machine-learning derived probability for a sample to be of low quality, as derived by the seqQscorer tool [11]. From 12 publicly available RNA-seq datasets, we downloaded a maximum number of 10 million reads per FASTQ file. Some quality features were derived from the full files and others from a subset of 1,000,000 reads (see Methods). Previously, we observed that random subsampling of the reads does not strongly impact the predictability of P_low_ and this significantly reduced computing time.

**Fig. 2:**
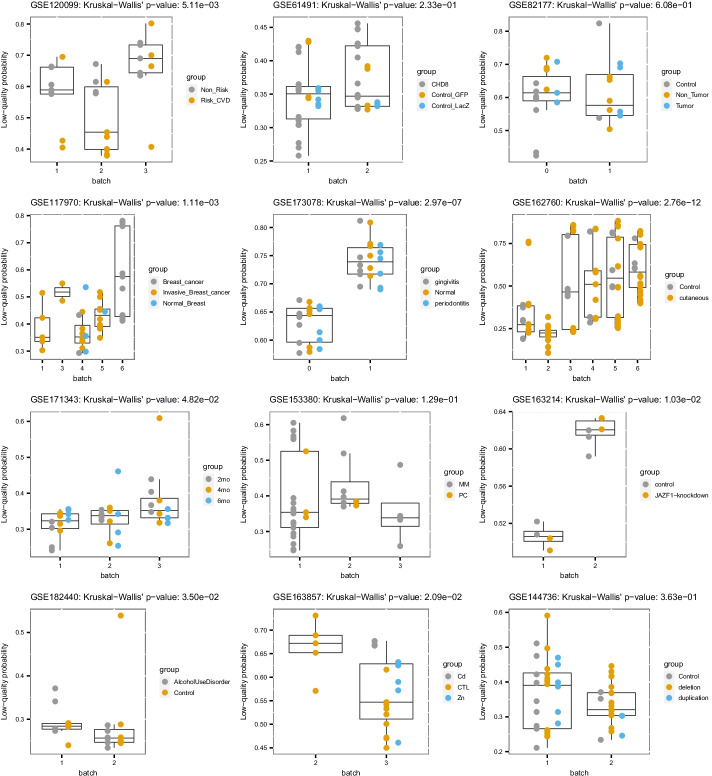
Predicted low-quality scores (P_low_) of all samples for each surveyed dataset. Shapes represent sample’s batches and colors represent sample’s groups. The p-values of a Kruskal–Wallis’s rank sum test of the batch against P_low_ is given in the title of each plot

In 6 datasets, the samples have a significant difference of P_low_ scores between the batches. In 5 datasets, the differences are not significant (Fig. 2). One dataset shows a marginally significant difference (close to the selected threshold of 0.05). Those results confirm the ability of the quality scores to detect batches in some datasets. For the datasets showing no significant difference, other experiments would be required to clarify if there are no substantial batch effects, if the batch effect is unrelated to quality, or if the method failed. For example, a batch effect that is not related to quality could still be observed by clustering analyses or by detailed analysis of dysregulated genes and related pathways.

**Fig. 3:**
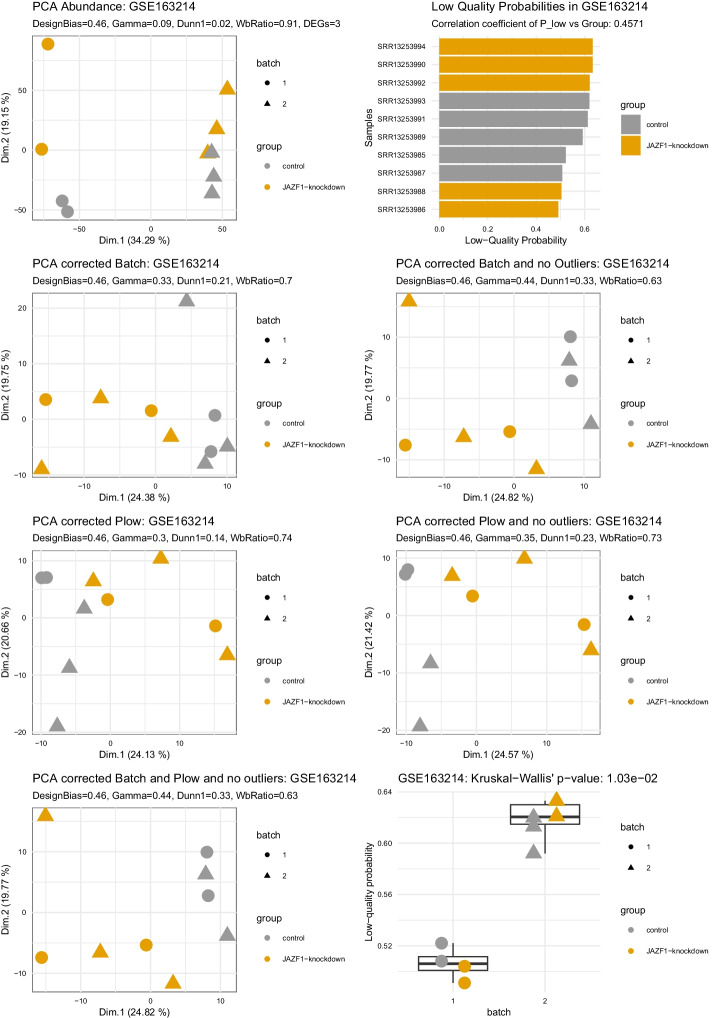
Expression data and four different types of batch correction. From top left to bottom right: PCA Abundance, shows the uncorrected PCA of the rlog normalized counts quantified by salmon and imported to a deseq2 object, next to it in the top right panel a bar plot shows the Low-Quality probability P_low_ for each sample. The PCA corrected with batch uses the AC-PCA package [21] to return principal components that were computed with the true batch as a confounding factor. The PCA corrected with P_low_ uses the AC-PCA package likewise, but P_low_ as a confounding factor. To the right of either corrected PCA we see a corrected PCA on the basis of the data without outliers. For the correction with the real batch, we removed outliers identified from either the base PCA or the corrected version. In P_low_ we also removed outliers based on the corrected PCA, but additionally added a threshold for P_low_ after manual inspection of the bar plot on the top right. The last two panels are the PCA corrected by both batch and P_low_ and the Boxplot of Batch against P_low_

Figure 3 shows detailed results for the dataset GSE163214. A strong difference in quality can be observed between the batches on the boxplot, supported by a significant Kruskal–Wallis’s test (p-value = 1.03e−2) and a high correlation coefficient of P_low_ vs sample’s groups (designBias = 0.44; also illustrated by the bar plot). We can observe a strong batch effect in the uncorrected principal component analysis (PCA) (top row, left) where samples from batch 1 cluster together on the right-hand side, and samples from batch 2 on the left-hand side (panel: PCA Abundance). It is supported by poor clustering evaluation scores (the higher the better: Gamma = 0.09, Dunn1 = 0.01; the lower the better: WbRatio = 0.91) and very few differential genes (DEGs = 4). Thanks to the reference method using a priori knowledge of the batches to correct the analysis, samples on a batch-corrected PCA cluster by group and not anymore by batch (panel: PCA corrected Batch). It is supported by more differentially expressed genes (DEGs = 12) and better clustering evaluation scores (Gamma = 0.32, Dunn1 = 0.17; WbRatio = 0.68), with further improvement on another version of the batch-corrected PCA where an outlier sample could be manually identified and removed (sample: SRR13253993; panel: PCA corrected Batch and no outlier). Without using a priori knowledge but only using the automatically derived quality scores to correct the analysis, samples are also clustered by group and not anymore by batch (panel: PCA corrected P_low_). Clustering results and statistics are very comparable to the reference that uses a priori knowledge of the batches, though the number of differentially expressed genes is even higher (DEGs = 21) and removing outliers manually or based on quality scores did not improve the results further. Finally, applying a correction based on both a priori knowledge and automatic quality scores, together with outlier removal, showed the best clustering statistics (panel: PCA corrected Batch and P_low_ and no outlier) (Gamma = 0.49, Dunn1 = 0.31; WbRatio = 0.58).

**Fig. 4:**
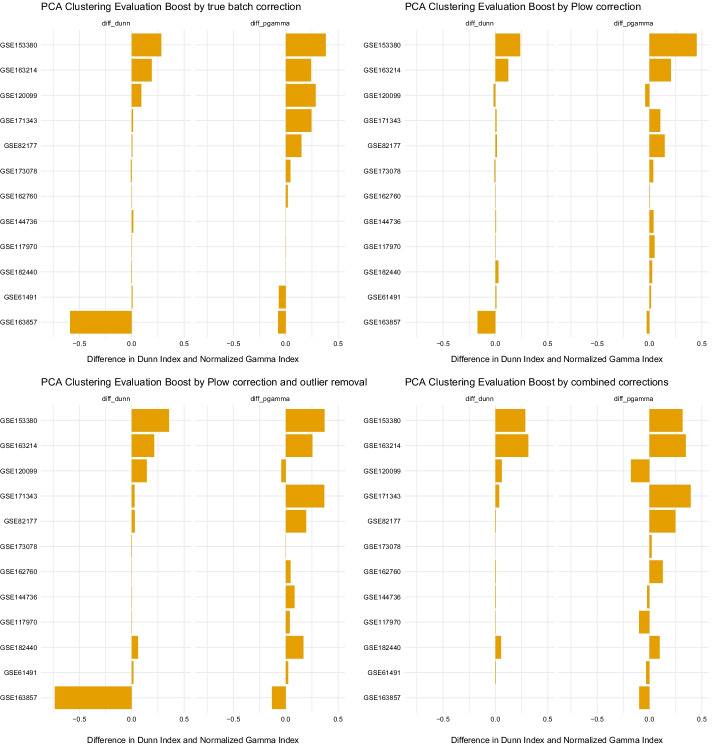
Difference in clustering metrics of the non-corrected PCA versus the batch and P_low_ corrected ones, as well as a PCA with removed outliers depending on P_low_. Quality-based correction can be compared to the true batch correction that is the reference method. The last plot on the bottom right shows the combination of P_low_ correction and removed outliers. Datasets are arranged in descending order by the difference of both values

In Fig. 4 the differences of clustering metrics for each dataset before and after correction are plotted. Across the datasets, the impact that batch correction and P_low_ correction have is mostly comparable, although the true batch correction seems to work better in a few cases (e.g. dataset GSE120099). However, if outliers of the base PCA or depending on sample quality are removed, the impact improves and even overcomes the simple batch correction. It should be noted that these clustering metrics are also sensitive to the removal of large proportions of samples, which is sometimes the case here. The impact of outlier removal alone is negligible (data not shown). All PCAs that are discussed here are given as panel plots similar to Fig. 3 in Additional file 1.

An additional view on the data is shown in Table 1. The table reports manual evaluation of the clustering results. Manual evaluation was necessary to overcome the limitations of the clustering metrics that do not handle biologically expected similarities between samples correctly. Taking the dataset GSE82177 as an example, clustering metrics would score poorly on the fact that Control and Non-tumor samples cluster together although it makes sense biologically. The two control groups of the dataset GSE61491 are another example. Also, the large negative changes to the clustering metrics after correction by the reference method or our approach for dataset GSE163857 were manually evaluated to be actually not significant and most likely due to a different scaling of the PCAs (the scale of the components shrinks two to three-fold; see Additional file 1).

**Table 1:** Manual exploration of the PCA plots with different corrections for all datasets

GEO series	Exp. design (group vs batch)	Design bias (P_low_ vs group)	Kruskal Wallis’s P-value (batch vs P_low_)	Batch effect on base PCA	Batch effect removed after batch correction	Batch effect removed after Plow correction	Plow performance compared to batch	Plow performance with outlier removal	Performance of combined correction
GSE120099	Good	0.655	4.24E−03	Yes	Yes	No	Worse	Worse	Worse
GSE117970	Poor	0.608	8.41E−04	Yes	No	Yes	Better	Better	Worse
GSE163857	Poor	0.522	2.09E−02	No	–	–	Comparable	Comparable	Worse
GSE162760	Good	0.496	2.36E−12	Yes	Yes	Yes	Comparable	Better	Comparabe
GSE182440	Very good	0.495	1.06E−01	No	–	–	Comparable	Better	Better
GSE144736	Poor	0.494	3.63E−01	Yes	Yes	No	Comparable	Better	Worse
GSE82177	Very good	0.493	5.75E−01	Yes	Yes	No	Comparable	Better	Better
GSE171343	Very good	0.488	8.25E−02	Yes	Yes	No	Comparable	Comparable	Comparable
GSE173078	Very good	0.479	2.93E−07	No	–	–	Comparable	Comparable	Comparable
GSE61491	Good	0.448	2.13E−01	Yes	Yes	No	Comparable	Comparable	Better
GSE163214	Good	0.443	1.03E−02	Yes	Yes	Yes	Comparable	Comparable	Better
GSE153380	Poor	0.442	1.58E−01	No	–	–	Comparable	Better	Better

Exp. Design is a manually given label evaluating the balance of the biological groups between the batches. Design Bias evaluates the clustering of the biological groups by quality scores P_low_ (normalized gamma of P_low_ against the group; the higher, the better the clustering by quality; values from 0 to 1). The Kruskal–Wallis’s P-value is derived from a Kruskal–Wallis’s test comparing average P_low_ values by batch. Taken together, those metrics show the potential association between our quality metric, groups, and batches. Other columns show the manual evaluation of the batch effect and correction methods

Overall, the P_low_ correction was mostly evaluated as comparable to or better than the reference method that uses a priori knowledge of the batches (in 10 and 1 of 12 datasets, respectively; total = 92%). When coupled to outlier removal, the P_low_ correction was more often evaluated as better than the reference (comparable or better in 5 and 6 datasets of 12, respectively; total = 92%). Combining true batch and P_low_ could improve the results further but not systematically (5 datasets better but also 4 worse than the best tested correction method (P_low_ or true batch)) and notably with a strong association with the imbalance of quality between groups of samples (designBias): the lower the bias, the better this further improvement. Although performing well, the P_low_ correction could not systematically remove the batch effect as well as the reference method using a priori knowledge. This result is in agreement with the multifaceted nature of batch effect, which is not only explained by quality differences.

However, in some cases it is clear that the observable batch effect is not related to quality. In these cases, the batch effect could be countered when correcting for the real batch but not with P_low_ (GSE120099) or vice versa a correction with P_low_ would improve the clustering, but batch correction would not (GSE117970) (Additional file 1). In GSE120099 we even observe a batch-like difference in quality between two groups of samples, which does not correspond to the actual annotated batches, but seems confounded with the group, resulting in the highest value for the Design bias.

Also, a good example for a divergence of batch effect and quality is dataset GSE82177: two very strong outliers skew the PCA plot and we observe a batch effect. When P_low_ or batch correction is employed, the PCA can be deconvoluted and the points are scattered as expected, but still intertwined. When removing the outliers, the batch correction is not able to differentiate between the groups well; with the quality-dependent outlier removal the clustering is better. However, when we use both P_low_ and true batch correction, the control and non-tumor samples cluster together and the tumor samples are loose but not close to the others.

In Additional file 2, panel plots similar to Additional file 1 are available for each dataset, showing the correction of the PCA plot with surrogate variables detected by SVA [9]. The first surrogate variable often has similar impact as either the true batch or P_low_, while the combination of all surrogate variables together can sometimes outperform the true batch as well as P_low_, suggesting artifacts of biological origin in the data: this is the case in GSE117970 and GSE171343, although in the latter P_low_ can achieve good results when removing an outlier. Vice versa, in some cases the correction with the combined surrogate variables skews the data, most likely, because relevant biological information was identified as an artifact, see GSE82177, GSE162760 and GSE153380. The latter is even skewed by just the first surrogate variable alone, which could be rooted in the unbalanced nature of the dataset.

Overall, although batch effects are not only explained by quality differences, quality-based data correction performs similarly to real batch correction on sample clustering.

## Discussion

In this work we have tested our automated quality analysis tool, seqQscorer, for its capabilities to detect batch effects in the data. Taken as a confounding factor to correct the data for the clustering of the samples, the quality evaluation led to results comparable to the reference method that uses the real batch information.

We observed that in half of the data there were significant differences in the P_low_ quality score between the batches. We could observe that P_low_ would often have similar effects as correcting for the true batch, but also observed a divergence between batch effect and P_low_: In some datasets, we would correct for P_low_, but the batch effect was still clear, even when we otherwise improved clustering.

It should be noted that even with the correction based on the true batch, the PCA clustering could not always be improved. In fact, only for half of the datasets, a true batch correction would improve the clustering. This may be for a variety of reasons, such as the fact that there may not actually be a significant biological difference between the groups of samples. For example, in GSE162760 we can successfully remove the batch effect with both P_low_ and true batch correction but the samples do not cluster better by biological group. Also, PCA plots are not always appropriate to observe small changes between samples such as changes due to a disease that impacts the expression of only a few genes.

When correcting with P_low_ we could observe similar levels of improvement for the clustering and even surpassed the batch correction when removing outliers. However, the selected metrics could be impacted by changes in the number of samples and not only by the clustering of the data points. Such metrics also do not weigh expected and non-expected biological group similarities differently. Nevertheless, the manual inspection of the plots leads to the same conclusion.

When combining the two methods (correcting based on a priori knowledge of the batches and automated quality control), we observed very different outcomes. The combination of the methods could perform better or worse in comparison to the individual approaches. We hypothesize that the improvement of a combination of two confounding factors depends on whether they model different artifacts in the data or not. We did not take into account other confounding factors, since they are generally not well documented in public datasets. Poor results in this study from true batch or quality-based correction could be explained by such unaccounted confounding factors.

Batch effects that would impact data quality, such as effects explained by different handlers, sequencers or reagents during RNA extraction, will most likely be detected by our software [1, 13]. Biological artifacts explained for example by the origin of the tissue or location of sampling would not impact quality, but they can still majorly skew the data [1]. Experiments to investigate sources for such biases, would be best addressed by the wet-lab in charge of the analysis: it would be necessary to perform an NGS experiment with deliberate batches. To have a possibility to observe if these batch effects are related to quality, the files would need to be evaluated by seqQscorer as well as manually by an experienced NGS scientist, for example the RNA Integrity Number (RIN) values should be used as an indicator. Another possibility to approach this would be to do the same experimental setup with batches two times, one time by an experienced handler and one time by a less experienced handler or by inducing errors that will likely impact the quality directly, like not keeping the temperature steady or gradually overreach the given time limits during library preparation.

Batch effect correction algorithms such as SVAseq and RUVseq can detect any types of artifacts in the data and could potentially outperform a simple correction by known batch or by a single confounding factor such as P_low_. However, they must be guided with information about the biological groups of interest beforehand in order to avoid their detection as unwanted variation to be corrected [4, 14]. Such detection methods bring the risk of detecting biologically relevant information, such as subpopulations in groups of the data, as bias and subsequently removing this information [8]. This was also observed in this work: while the sample-group-informed surrogate variable could often outperform P_low_ or even the true batch information, it could be observed that in 25% of the cases investigated the correction would skew or even remove the group clustering (see Additional file 2). Goh et al. suggest to use a combination of gene fuzzy scoring with neural-network-based feature extraction to circumvent this problem for downstream application without data transformation, especially with machine learning applications in mind [8]. Future work could explore ensemble methods leveraging a variety of models specializing in different technical effects or artifacts, together with our quality-aware models.

Overall, our automated quality control method produces a score able to model technical differences between the samples of a dataset. It can be used as a confounding factor in downstream analysis. However, there are still biological artifacts and batch effects that are not explained by quality. It would be desirable to increase the number of datasets to be able to better investigate if there are properties in the data and metadata that give rise to artifacts. Cross referencing this information to batch correction and correction by P_low_ could give insight about the origins of artifacts in the data. Still, this type of information is not commonly given in publicly available datasets. Even with regard to batches, it is not easy to find well annotated datasets, which led to the small number of them used in this work.

## Conclusions

In this work, we used our machine learning software that estimates the probability of low quality for a given RNA-seq, DNAse-Seq or ChIP-Seq sample and showed that the produced probability score P_low_ can be used to predict batch effects that arise from technical issues.

We were also able to use the score to aid clustering visualization of the data and to remove the batch effect comparably to the reference method that uses a priori knowledge. We observed the existence of batch effects that our quality-based approach could not identify and most likely stemmed from biological artifacts.

## Methods

We used 12 batched RNA-seq datasets from the NCBI’s GEO database. Table 2 shows the datasets that were used and gives some metrics about the data. 7 batched datasets were paired-end and 5 datasets single-end RNA-sequencing. The smallest number of samples in a dataset is 10 samples, the highest number is 128. Metadata was collected by hand from GEO and SRA and the datasets were downloaded using the samples’ SRA accession numbers by the fastq-dump tool from the SRAtools library (10,000,000 random reads were selected for download with the -X flag). The computation was done on a personal computer with an Intel® Core™ i7-8700 K CPU and 32 GB ram. With 5 threads, the genome mapping process for a FASTQ file was between 20 and 30 s. The script that derives LOC and TSS features took approximately 40 s per file.

**Table 2:** Batched datasets used in this work

Dataset	Authors	Tissue	Disease	n samples	n groups	n batches	Sample filtering
GSE120099	Lo Sardo et al. [24]	Vascular smooth muscle cells	Cardiovascular diseases	29	2	3	Only WT cells
GSE61491	Sugathan et al. [25]	Neural progenitor cells	Memory disorders	54	2 (3)	2	No
GSE82177	Wijetunga et al. [26]	Liver	Carcinoma, hepatocellular	27	3	2	No
GSE117970	Cassetta et al. [27]	Breast	Breast neoplasms	53	3	5	Only breast related samples
GSE173078	Hyunijin Kim et al. [28]	Periodontium	Periodontitis, gingivitis	36	3	2	No
GSE162760	Farias et al. [29]	Whole blood	Leishmaniasis, cutaneous	128	2	6	No
GSE171343	Bowles et al. [30]	IPSC derived cerebral organoids	Dementia	36	3	3	Only of one Type: GIH-6-C1-(delta)A02
GSE153380	Alvarez-Benayas et al. [31]	Primary plasma—and myeloma cells	Muliple myeloma	33	2	3	Only primary cells
GSE163214	Procida et al. [32]	HeLa Kyoto cell line	None	10	2	2	No
GSE182440	Lim et al. [33]	Brain	Alcoholism	24	2	2	No
GSE163857	Moser et al. [34]	Microglia		24	3	2	Only human samples
GSE144736	Roth et al. [35]	iPSC-derived patient neuroepithelium	Microcephaly	52	3	2	No

Samples in each dataset have been filtered to remove factors that could bias batch effect evaluation

Most datasets are related to diseases, except for dataset GSE163857, which is surveying the effects of metals on Microglia cells, and GSE163214 surveying the impact of a gene on overall gene expression and histone acetylation in a HeLa cell line. Surveyed diseases in the datasets span from neuronal diseases (e.g. Microcephaly, Dementia or the impact of Alcoholism on neuronal cells) to inflammatory diseases (e.g. periodontitis and parasitic diseases with leishmaniasis) and also different cancer types and cardiovascular diseases, showing a broad overview over biological data despite the relatively small number of datasets. Table 2 gives an overview of the datasets and the corresponding publications. As indicated in the last row, for some datasets only a subset of the samples was chosen, either to keep the number of samples small, or if the samples were too convoluted to get any cluster metrics.

We used our software seqQscorer to derive the quality features and to predict the quality of a sample, assigning the probability of low-quality P_low_ [11]. Our software first needs to derive the features from the FastQ files representing the biological samples. To this end, it utilizes four bioinformatic tools: FastQC to produce the RAW features (Summary statistics of FastQC) [15], Bowtie2 for the MAP features (genome mapping statistics) [16], ChIPSeeker for the LOC feature (percentages of reads in genomic regions) [17] and ChIPPeakAnno for the TSS features (percentages of reads in bins around the transcription start sites) [18]. FastQC was applied to the full downloaded FastQ file, while other quality features were derived from 1,000,000 randomly selected reads. The tool uses these features to compute the probability score P_low_ for each sample. Among the predefined models of seqQscorer some were trained on certain subsets of a large main training set: 2642 samples labeled for low- and high-quality. We used the generic Random Forest model that was trained on the complete training set. With this generic model, P_low_ is computed from the ratio of votes by trees in the random forest. To derive the differential gene expression, we used Salmon [19] to quantify the gene expression from 1,000,000 randomly selected reads per sample, and DESeq2 [20] for statistical tests and to compute rlog normalized expression values for PCA plotting. Differentially expressed genes were considered at an adjusted p-value < 0.05. See Fig. 1 for an overview of the computing workflow.

We plotted the batches against P_low_ with R and ggplot2 and computed a Kruskal–Wallis’s test to confirm if there are significant differences of P_low_ values between batches.

To investigate if P_low_ values could be used for batch corrections, we observed the first two dimensions of a PCA either without any correction, or with correction by P_low_ as well as correction by the real batch identifier as confounding factors. We used the AC-PCA package for this, which allows to add confounding factors to the computation of principal components [21]. We also tested the impact of removing outliers according to P_low_ as well as the combination of all correction methods and outlier removal. Outliers removed according to batch correction were identified by the uncorrected PCA as well as the PCA with correction by the true batch. If a sample or a small group of samples was clearly skewing the clustering it was removed. The same was done for the P_low_ correction, with the help of the bar plot showing P_low_ values by samples (a sudden increase in high values was considered as a potential threshold to identify outliers). For the combination of all outliers, the outliers of P_low_ were used, since they always contained the outliers used for the batch correction and normal PCA. To have a metric of evaluation for these plots, we used the clusterstats function from the fpc package and employed the wbratio, pearsongamma and dunn1 statistics [22].

The dunn statistic considers the ratio of the minimum separation of all clusters by the maximum diameter of all clusters. That makes it a worst-case indicator, since it shows the smallest index, even if some of the clusters are well defined and only one cluster is scattered.

The pearsongamma is the correlation between distances and a 0–1-vector in which 0 indicates the same cluster, while 1 indicates a different cluster. It is derived from the Normalized gamma of Halkidi et al. [23].

The wbratio is the average distance within the clusters divided by the average distance between the clusters. While the other two metrics increase with clustering improvement, wbratio decreases.

In Table 1 we give a manual evaluation of the PCA plots: “comparable” means that a correction with P_low_ (either with or without outliers) worked similarly to a batch correction (with or without outliers, respectively). The same information is given for the combination of both corrections, but the reference is then the best overall PCA plot. One main point considered was the change of the grouping of the clusters from the second to the first principal component and if the groups clustered together. Especially a widely scattered cluster which can often be found with cancer versus normal tissue would not score high with the metrics used, but it would be easily identified at a glance. It is furthermore indicated if the experimental design is poor, good, or very good, according to the manual observation of the balance of samples between batches. If there is information about confounding factors available, it is also taken into account.

We computed a design bias representing the agreement of P_low_ to biological groups, utilizing Pearson gamma or “normalized gamma” [23], to have a positive value between zero and one we added one and divided the result by two.

We compared our results with the results of the sva method of the package of the same name [9]. We used it according to its documentation and used the biological group information and the expression data. Other possible confounding factors were not used.

## Supplementary Information

**Additional file 1.** Results of Correction Experiments for all Datasets.

**Additional file 2.** Comparison of P_low_ and SVA Correction.

## Data Availability

All data used in this work is publicly available via the NCBI GEO Database, all Accessions can be found in Table 2.

## Abbreviations

DEGs: Differentially expressed genes
NGS: Next-generation sequencing
PCA: Principal component analysis
RIN: RNA integrity number
SVA: Surrogate variable analysis
