# Supplementary material for: Batch effect detection and correction in RNA-seq data using machine-learning-based automated assessment of quality
Source: BMC Bioinformatics. 2022 Jul 14;23(Suppl 6):279. doi: 10.1186/s12859-022-04775-y (PMC9284682; doi:10.1186/s12859-022-04775-y)
Supplement: Supplementary file 1 — Additional file 1. Results of Correction Experiments for all Datasets. [file 12859_2022_4775_MOESM1_ESM.pdf]

Supplementary material S1

From top left to bottom right: PCA Abundance, shows the uncorrected PCA of the rlog normalized counts quantified by salmon and imported to a deseq2 object, next to it in the top right panel a bar plot shows the Low-Quality probability P_{low} for each sample. The PCA corrected with batch uses the acPCA package to return principal components that were computed with the true batch as a confounding factor. The PCA corrected with P_{low} uses the acPCA package likewise, but P_{low} as a confounding factor. To the right of either corrected PCA we see a corrected PCA on the basis of the data without outliers. For the correction with the real batch, we removed outliers that showed up either in the base PCA or in the corrected version. In P_{low} we also removed outliers of base or corrected PCA, but additionally added a threshold for P_{low} depending on the barplot on the top right. The last two panels are the PCA corrected by both batch and P_{low} and the Boxplot of Batch against P_{low}.

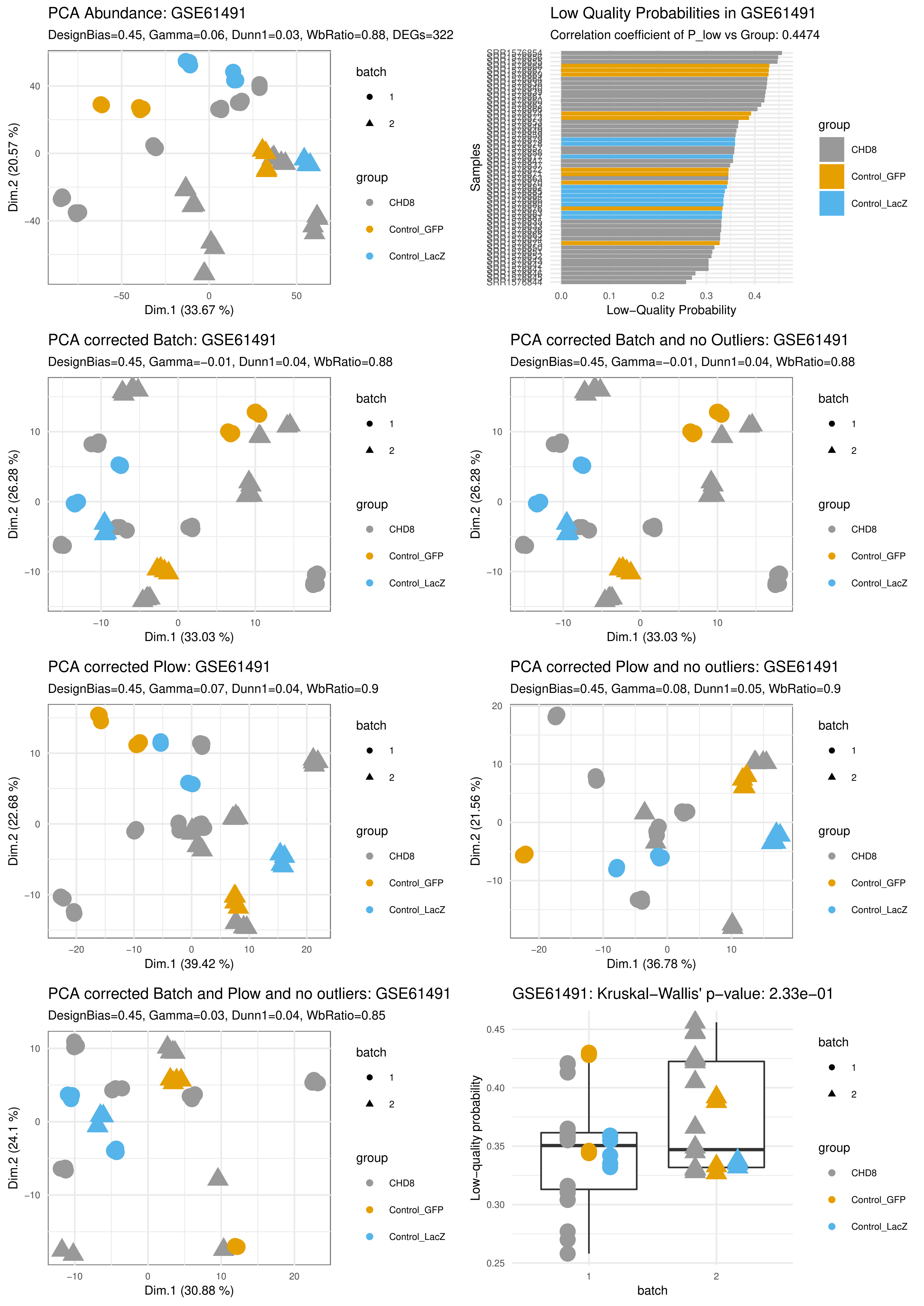
PCA Abundance: GSE61491
DesignBias=0.45, Gamma=0.06, Dunn1=0.03, WbRatio=0.88, DEGs=322
Dim.1 (33.67 %)
Dim.2 (20.57 %)
Low Quality Probabilities in GSE61491
Correlation coefficient of P_low vs Group: 0.4474
Samples
Low−Quality Probability
PCA corrected Batch: GSE61491
DesignBias=0.45, Gamma=−0.01, Dunn1=0.04, WbRatio=0.88
Dim.1 (33.03 %)
Dim.2 (26.28 %)
PCA corrected Batch and no Outliers: GSE61491
DesignBias=0.45, Gamma=−0.01, Dunn1=0.04, WbRatio=0.88
Dim.1 (33.03 %)
Dim.2 (26.28 %)
PCA corrected Plow: GSE61491
DesignBias=0.45, Gamma=0.07, Dunn1=0.04, WbRatio=0.9
Dim.1 (39.42 %)
Dim.2 (22.68 %)
PCA corrected Plow and no outliers: GSE61491
DesignBias=0.45, Gamma=0.08, Dunn1=0.05, WbRatio=0.9
Dim.1 (36.78 %)
Dim.2 (21.56 %)
PCA corrected Batch and Plow and no outliers: GSE61491
DesignBias=0.45, Gamma=0.03, Dunn1=0.04, WbRatio=0.85
Dim.1 (30.88 %)
Dim.2 (24.1 %)
GSE61491: Kruskal−Wallis' p−value: 2.33e−01
Low−quality probability
batch
batch
1
2
group
CHD8
Control_GFP
Control_LacZ

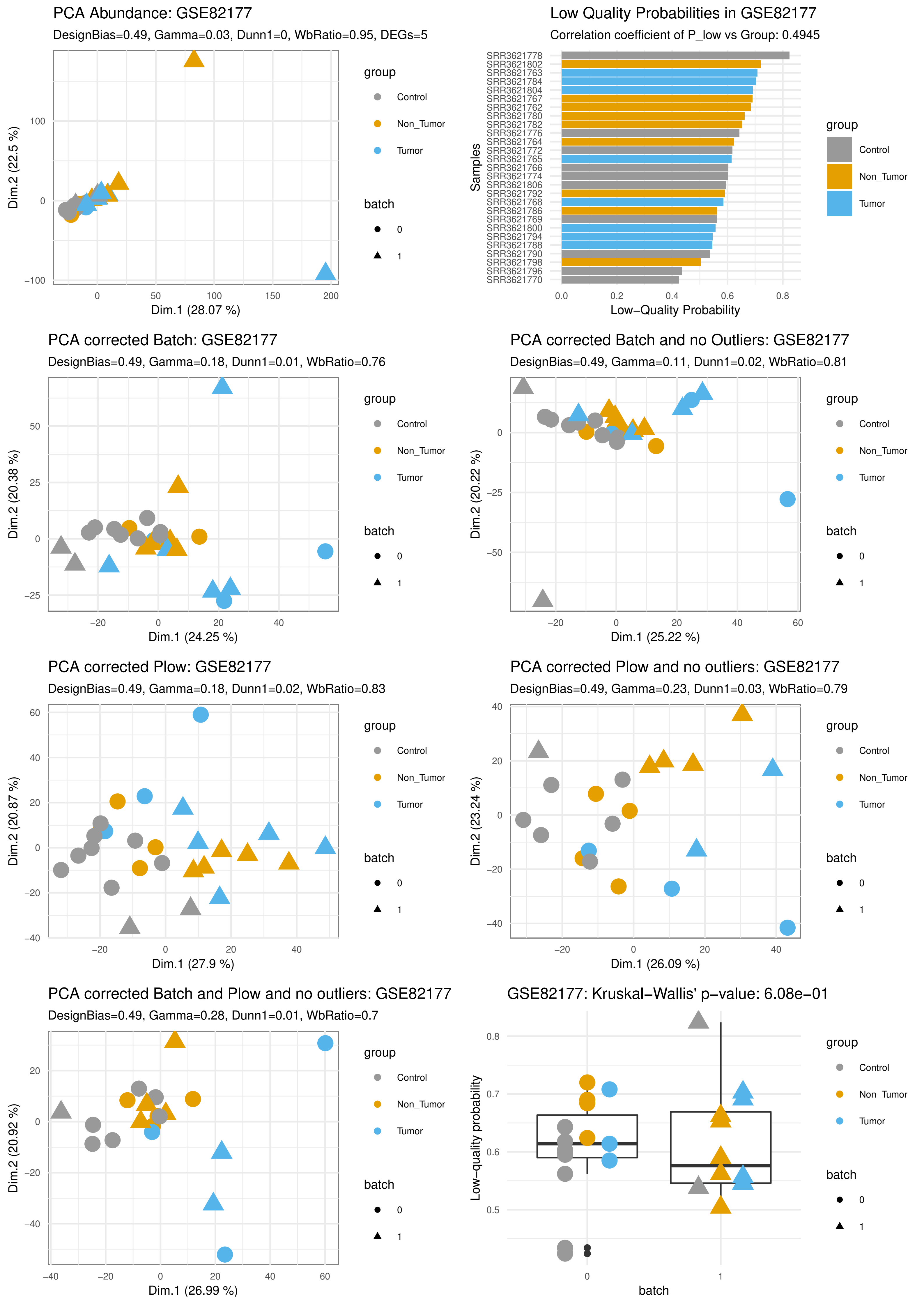
PCA Abundance: GSE82177
DesignBias=0.49, Gamma=0.03, Dunn1=0, WbRatio=0.95, DEGs=5
Dim.1 (28.07 %)
Dim.2 (22.5 %)
group
Control
Non_Tumor
Tumor
batch
0
1
Low Quality Probabilities in GSE82177
Correlation coefficient of P_low vs Group: 0.4945
Samples
SRR3621778
SRR3621802
SRR3621763
SRR3621784
SRR3621804
SRR3621767
SRR3621762
SRR3621780
SRR3621782
SRR3621776
SRR3621764
SRR3621772
SRR3621765
SRR3621766
SRR3621774
SRR3621806
SRR3621792
SRR3621768
SRR3621786
SRR3621769
SRR3621800
SRR3621794
SRR3621788
SRR3621790
SRR3621798
SRR3621796
SRR3621770
Low−Quality Probability
PCA corrected Batch: GSE82177
DesignBias=0.49, Gamma=0.18, Dunn1=0.01, WbRatio=0.76
Dim.1 (24.25 %)
Dim.2 (20.38 %)
PCA corrected Batch and no Outliers: GSE82177
DesignBias=0.49, Gamma=0.11, Dunn1=0.02, WbRatio=0.81
Dim.1 (25.22 %)
Dim.2 (20.22 %)
PCA corrected Plow: GSE82177
DesignBias=0.49, Gamma=0.18, Dunn1=0.02, WbRatio=0.83
Dim.1 (27.9 %)
Dim.2 (20.87 %)
PCA corrected Plow and no outliers: GSE82177
DesignBias=0.49, Gamma=0.23, Dunn1=0.03, WbRatio=0.79
Dim.1 (26.09 %)
Dim.2 (23.24 %)
PCA corrected Batch and Plow and no outliers: GSE82177
DesignBias=0.49, Gamma=0.28, Dunn1=0.01, WbRatio=0.7
Dim.1 (26.99 %)
Dim.2 (20.92 %)
GSE82177: Kruskal−Wallis' p−value: 6.08e−01
Low−quality probability
batch

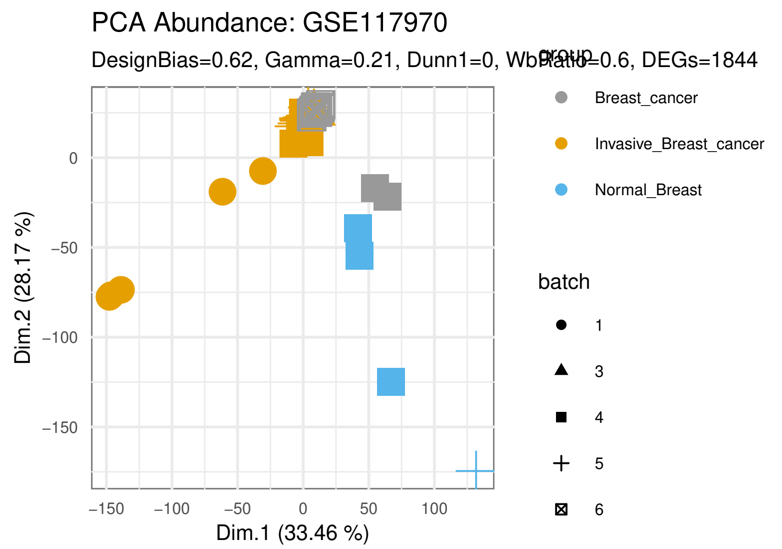
PCA Abundance: GSE117970
DesignBias=0.62, Gamma=0.21, Dunn1=0, WbRatio=0.6, DEGs=1844
Dim.1 (33.46 %)
Dim.2 (28.17 %)
group
Breast_cancer
Invasive_Breast_cancer
Normal_Breast
batch
1
3
4
5
6

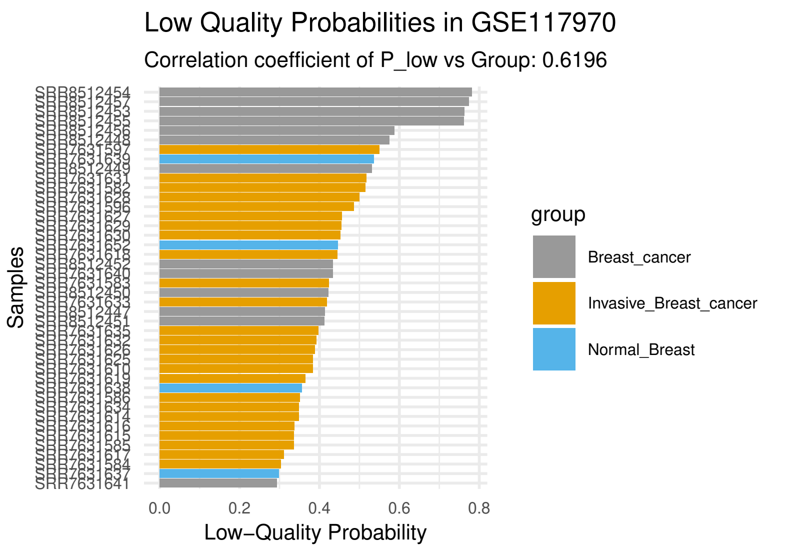
Low Quality Probabilities in GSE117970
Correlation coefficient of P_low vs Group: 0.6196
Samples
Low−Quality Probability
group
Breast_cancer
Invasive_Breast_cancer
Normal_Breast

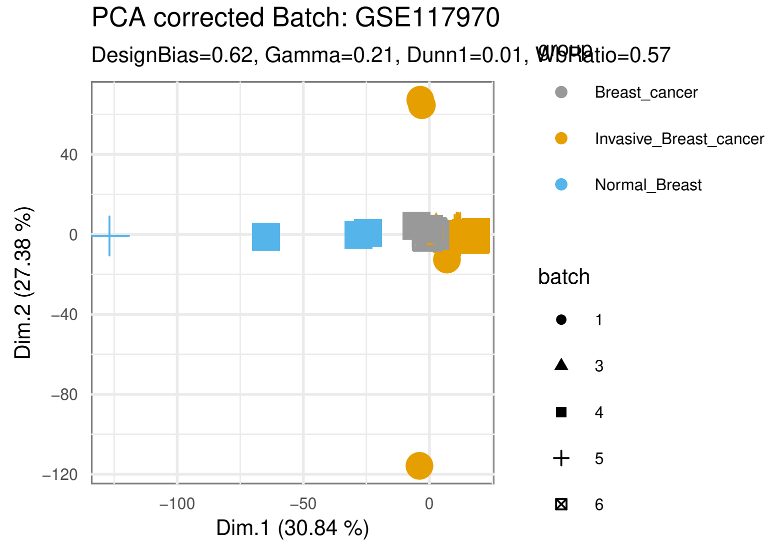
PCA corrected Batch: GSE117970
DesignBias=0.62, Gamma=0.21, Dunn1=0.01, WbRatio=0.57
Dim.1 (30.84 %)
Dim.2 (27.38 %)
group
Breast_cancer
Invasive_Breast_cancer
Normal_Breast
batch
1
3
4
5
6

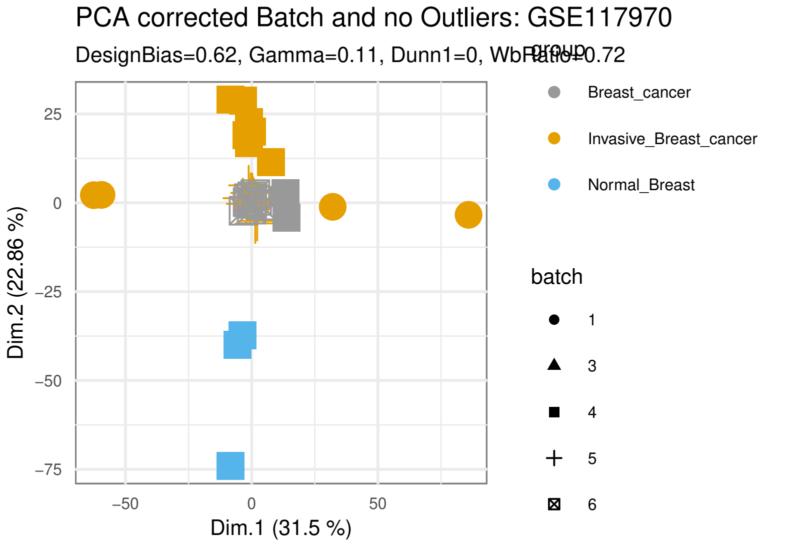
PCA corrected Batch and no Outliers: GSE117970
DesignBias=0.62, Gamma=0.11, Dunn1=0, WbRatio=0.72
Dim.1 (31.5 %)
Dim.2 (22.86 %)
group
Breast_cancer
Invasive_Breast_cancer
Normal_Breast
batch
1
3
4
5
6

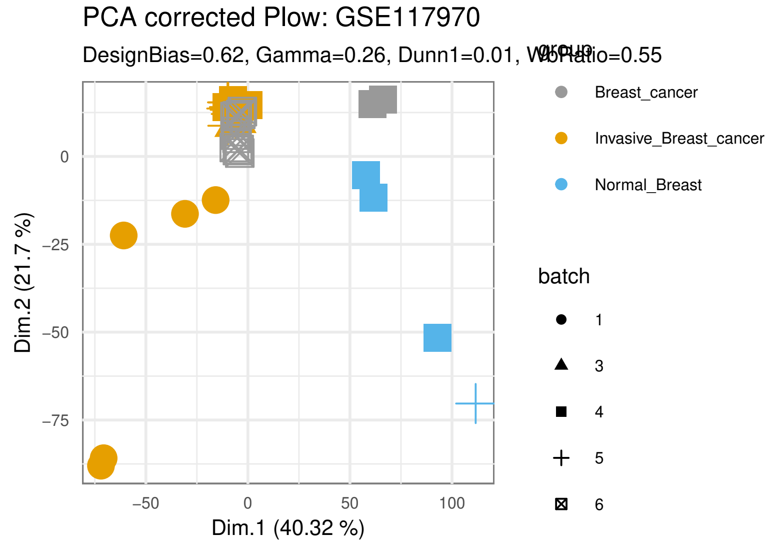
PCA corrected Plow: GSE117970
DesignBias=0.62, Gamma=0.26, Dunn1=0.01, WbRatio=0.55
Dim.1 (40.32 %)
Dim.2 (21.7 %)
group
Breast_cancer
Invasive_Breast_cancer
Normal_Breast
batch
1
3
4
5
6

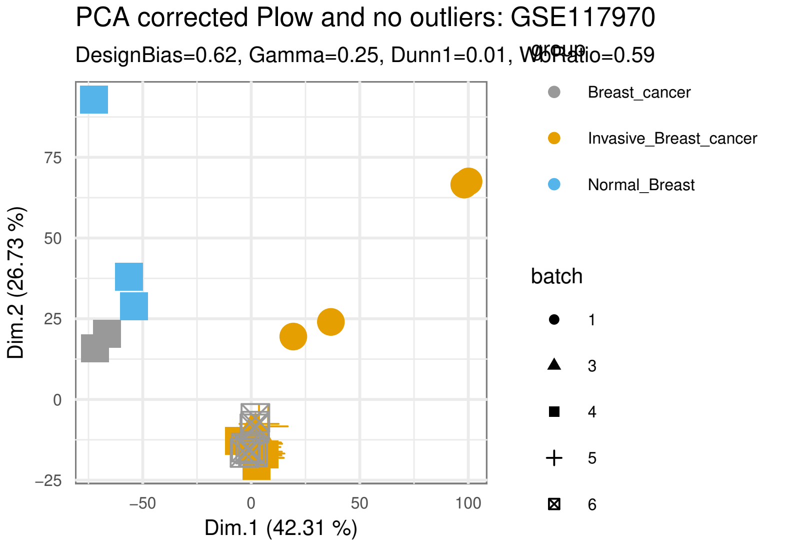
PCA corrected Plow and no outliers: GSE117970
DesignBias=0.62, Gamma=0.25, Dunn1=0.01, WbRatio=0.59
Dim.1 (42.31 %)
Dim.2 (26.73 %)
group
Breast_cancer
Invasive_Breast_cancer
Normal_Breast
batch
1
3
4
5
6

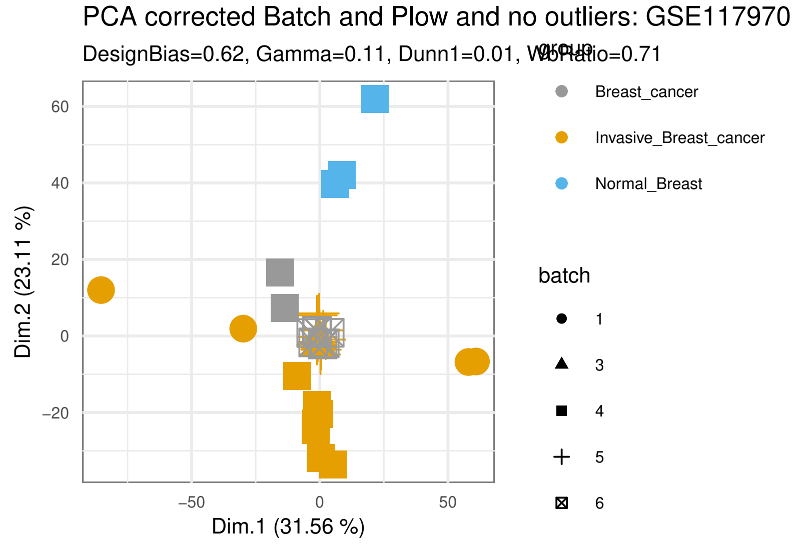
PCA corrected Batch and Plow and no outliers: GSE117970
DesignBias=0.62, Gamma=0.11, Dunn1=0.01, WbRatio=0.71
Dim.1 (31.56 %)
Dim.2 (23.11 %)
group
Breast_cancer
Invasive_Breast_cancer
Normal_Breast
batch
1
3
4
5
6

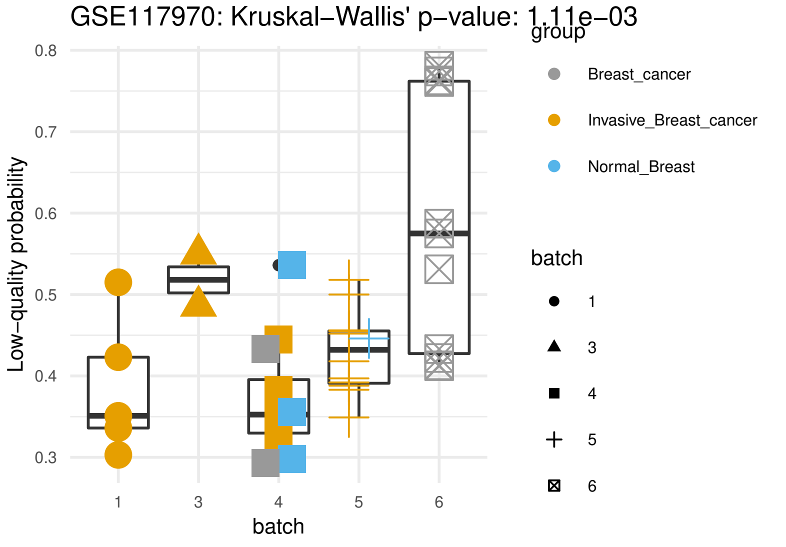
GSE117970: Kruskal−Wallis' p−value: 1.11e−03
Low−quality probability
batch
group
Breast_cancer
Invasive_Breast_cancer
Normal_Breast
batch
1
3
4
5
6

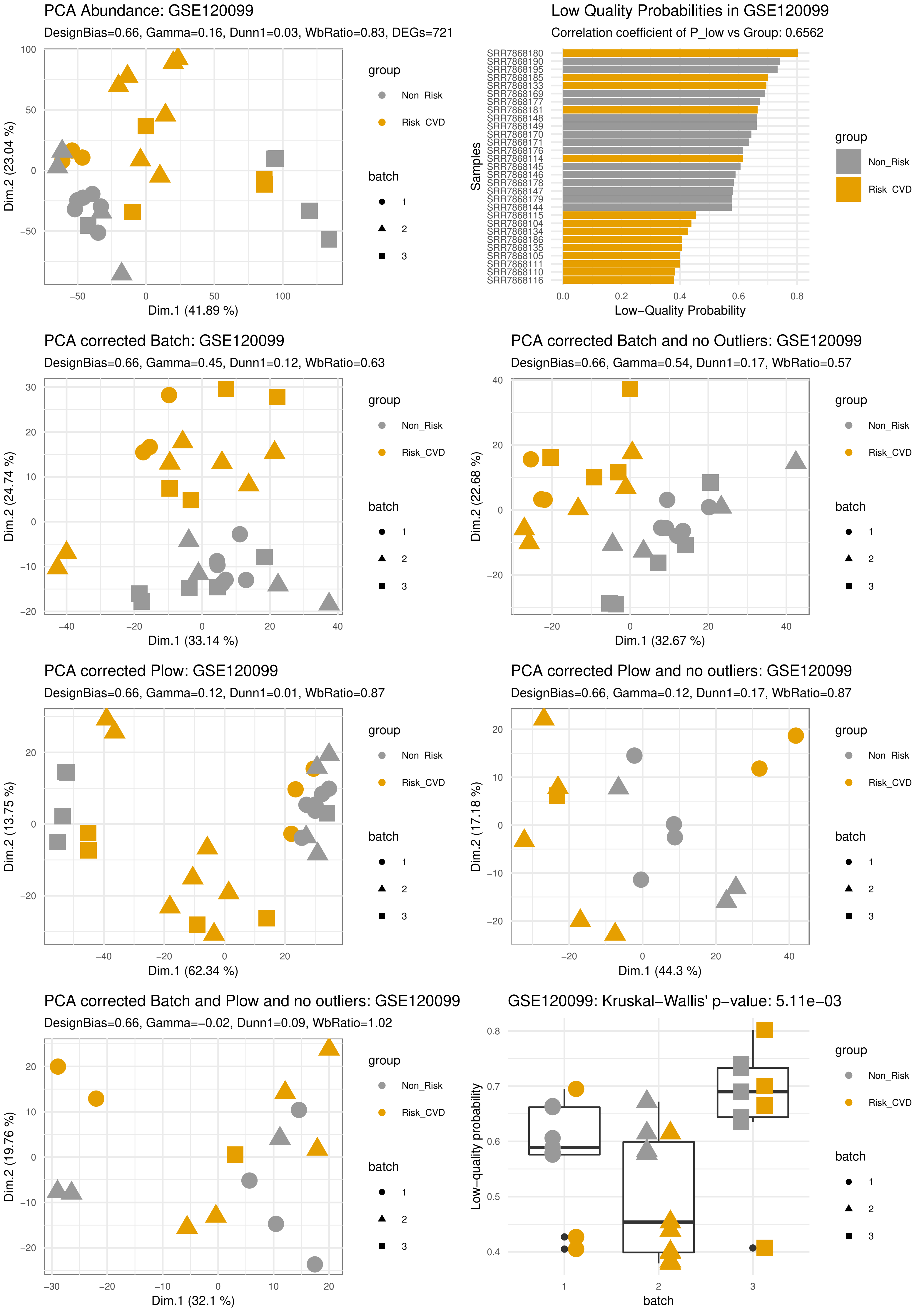
PCA Abundance: GSE120099
DesignBias=0.66, Gamma=0.16, Dunn1=0.03, WbRatio=0.83, DEGs=721
Dim.1 (41.89 %)
Dim.2 (23.04 %)
group
Non_Risk
Risk_CVD
batch
1
2
3
Low Quality Probabilities in GSE120099
Correlation coefficient of P_low vs Group: 0.6562
Samples
SRR7868180
SRR7868190
SRR7868195
SRR7868185
SRR7868133
SRR7868169
SRR7868177
SRR7868181
SRR7868148
SRR7868149
SRR7868170
SRR7868171
SRR7868176
SRR7868114
SRR7868145
SRR7868146
SRR7868178
SRR7868147
SRR7868179
SRR7868144
SRR7868115
SRR7868104
SRR7868134
SRR7868186
SRR7868135
SRR7868105
SRR7868111
SRR7868110
SRR7868116
Low−Quality Probability
PCA corrected Batch: GSE120099
DesignBias=0.66, Gamma=0.45, Dunn1=0.12, WbRatio=0.63
Dim.1 (33.14 %)
Dim.2 (24.74 %)
PCA corrected Batch and no Outliers: GSE120099
DesignBias=0.66, Gamma=0.54, Dunn1=0.17, WbRatio=0.57
Dim.1 (32.67 %)
Dim.2 (22.68 %)
PCA corrected Plow: GSE120099
DesignBias=0.66, Gamma=0.12, Dunn1=0.01, WbRatio=0.87
Dim.1 (62.34 %)
Dim.2 (13.75 %)
PCA corrected Plow and no outliers: GSE120099
DesignBias=0.66, Gamma=0.12, Dunn1=0.17, WbRatio=0.87
Dim.1 (44.3 %)
Dim.2 (17.18 %)
PCA corrected Batch and Plow and no outliers: GSE120099
DesignBias=0.66, Gamma=−0.02, Dunn1=0.09, WbRatio=1.02
Dim.1 (32.1 %)
Dim.2 (19.76 %)
GSE120099: Kruskal−Wallis' p−value: 5.11e−03
Low−quality probability
batch

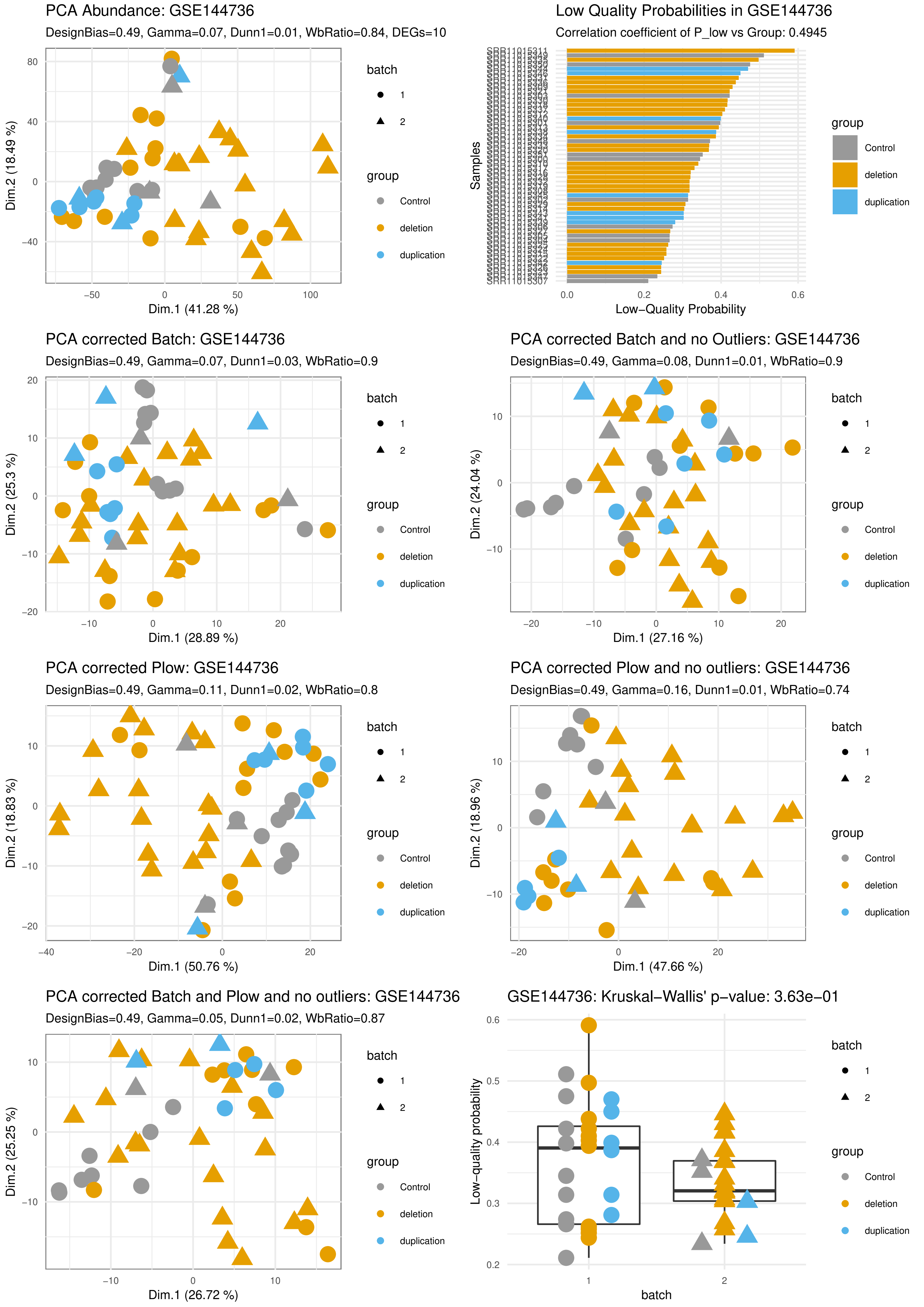
PCA Abundance: GSE144736
DesignBias=0.49, Gamma=0.07, Dunn1=0.01, WbRatio=0.84, DEGs=10
Dim.1 (41.28 %)
Dim.2 (18.49 %)
Low Quality Probabilities in GSE144736
Correlation coefficient of P_low vs Group: 0.4945
Samples
Low−Quality Probability
PCA corrected Batch: GSE144736
DesignBias=0.49, Gamma=0.07, Dunn1=0.03, WbRatio=0.9
Dim.1 (28.89 %)
Dim.2 (25.3 %)
PCA corrected Batch and no Outliers: GSE144736
DesignBias=0.49, Gamma=0.08, Dunn1=0.01, WbRatio=0.9
Dim.1 (27.16 %)
Dim.2 (24.04 %)
PCA corrected Plow: GSE144736
DesignBias=0.49, Gamma=0.11, Dunn1=0.02, WbRatio=0.8
Dim.1 (50.76 %)
Dim.2 (18.83 %)
PCA corrected Plow and no outliers: GSE144736
DesignBias=0.49, Gamma=0.16, Dunn1=0.01, WbRatio=0.74
Dim.1 (47.66 %)
Dim.2 (18.96 %)
PCA corrected Batch and Plow and no outliers: GSE144736
DesignBias=0.49, Gamma=0.05, Dunn1=0.02, WbRatio=0.87
Dim.1 (26.72 %)
Dim.2 (25.25 %)
GSE144736: Kruskal−Wallis' p−value: 3.63e−01
Low−quality probability
batch
1
2
group
Control
deletion
duplication

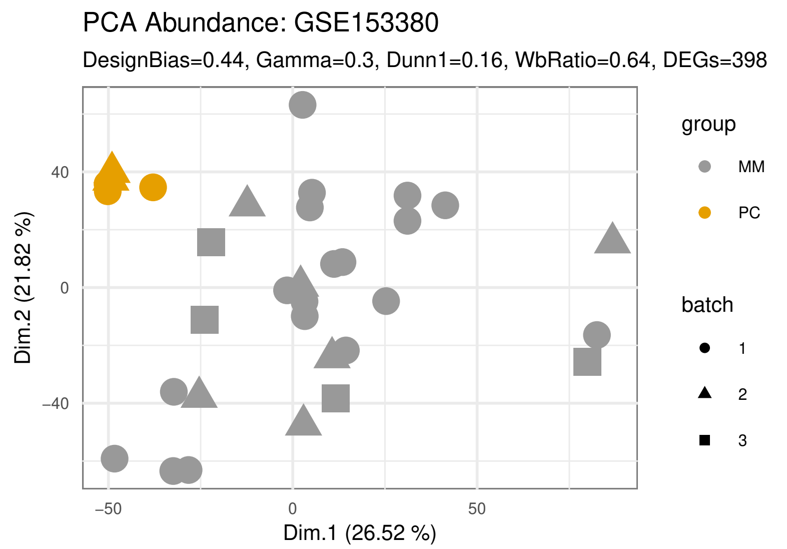
PCA Abundance: GSE153380
DesignBias=0.44, Gamma=0.3, Dunn1=0.16, WbRatio=0.64, DEGs=398
Dim.1 (26.52 %)
Dim.2 (21.82 %)
group
MM
PC
batch
1
2
3

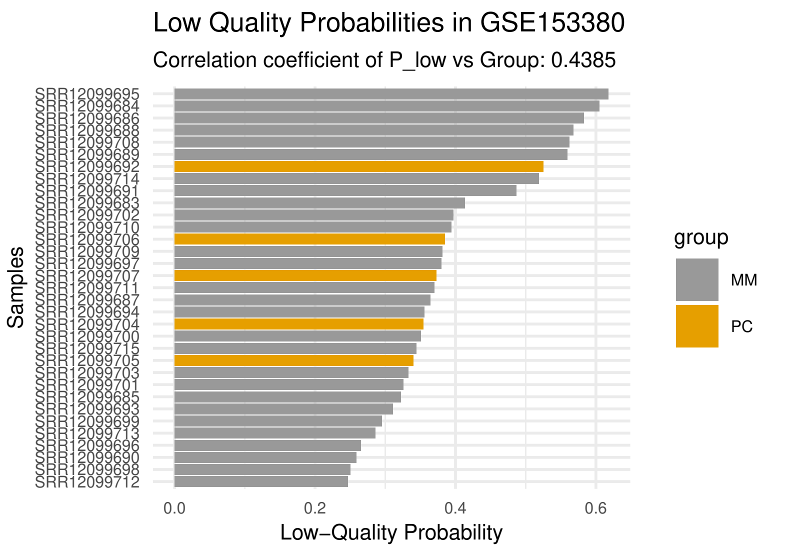
Low Quality Probabilities in GSE153380
Correlation coefficient of P_low vs Group: 0.4385
Samples
SRR12099695
SRR12099684
SRR12099686
SRR12099688
SRR12099708
SRR12099689
SRR12099692
SRR12099714
SRR12099691
SRR12099683
SRR12099702
SRR12099710
SRR12099706
SRR12099709
SRR12099697
SRR12099707
SRR12099711
SRR12099687
SRR12099694
SRR12099704
SRR12099700
SRR12099715
SRR12099705
SRR12099703
SRR12099701
SRR12099685
SRR12099693
SRR12099699
SRR12099713
SRR12099696
SRR12099690
SRR12099698
SRR12099712
Low−Quality Probability
group
MM
PC

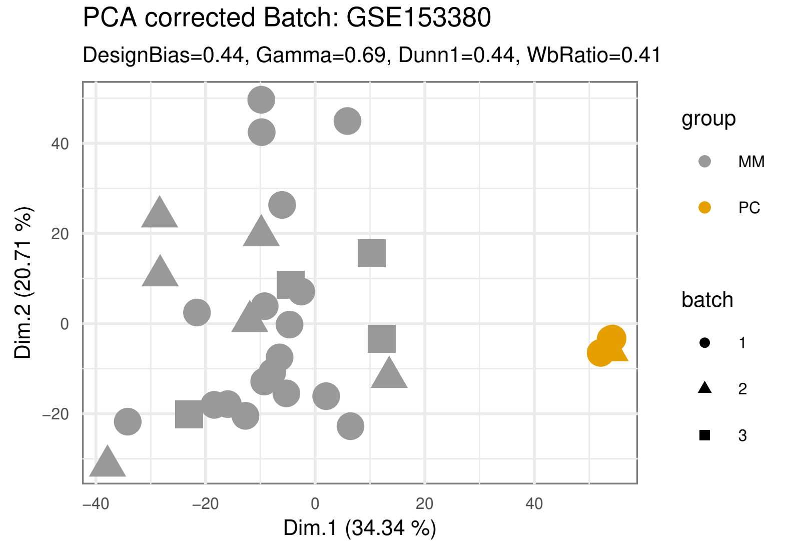
PCA corrected Batch: GSE153380
DesignBias=0.44, Gamma=0.69, Dunn1=0.44, WbRatio=0.41
Dim.1 (34.34 %)
Dim.2 (20.71 %)
group
MM
PC
batch
1
2
3

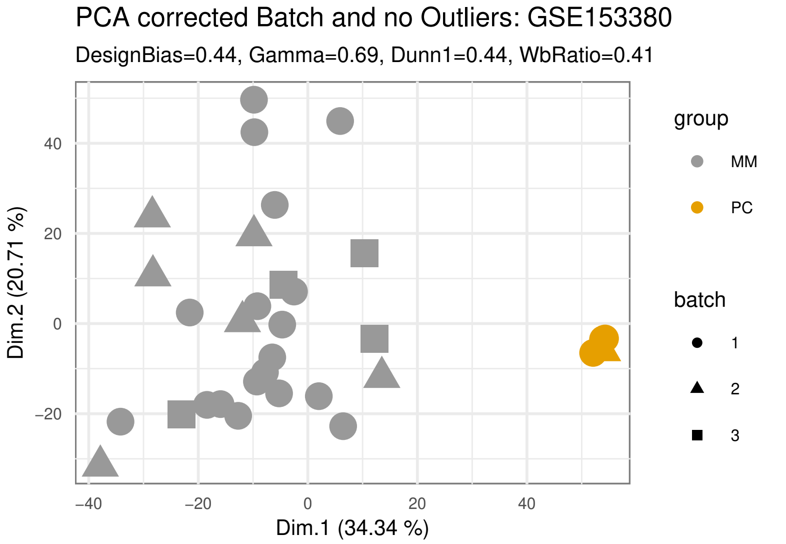
PCA corrected Batch and no Outliers: GSE153380
DesignBias=0.44, Gamma=0.69, Dunn1=0.44, WbRatio=0.41
Dim.1 (34.34 %)
Dim.2 (20.71 %)
group
MM
PC
batch
1
2
3

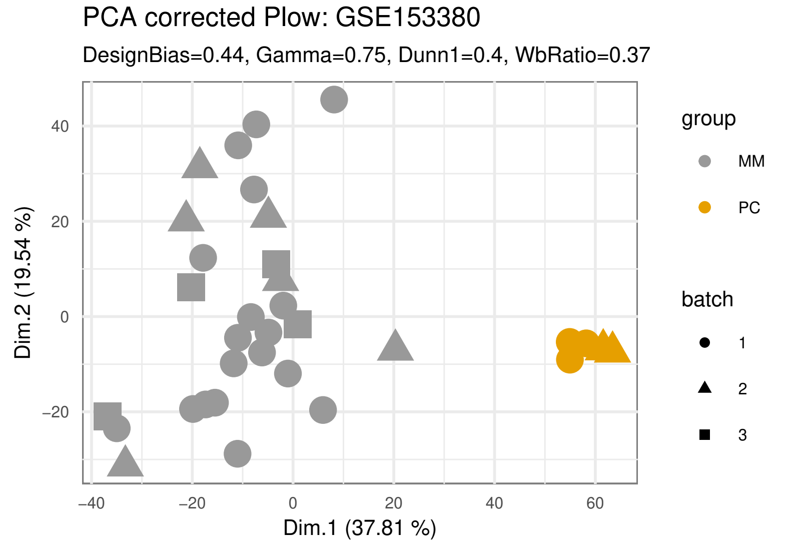
PCA corrected Plow: GSE153380
DesignBias=0.44, Gamma=0.75, Dunn1=0.4, WbRatio=0.37
Dim.1 (37.81 %)
Dim.2 (19.54 %)
group
MM
PC
batch
1
2
3

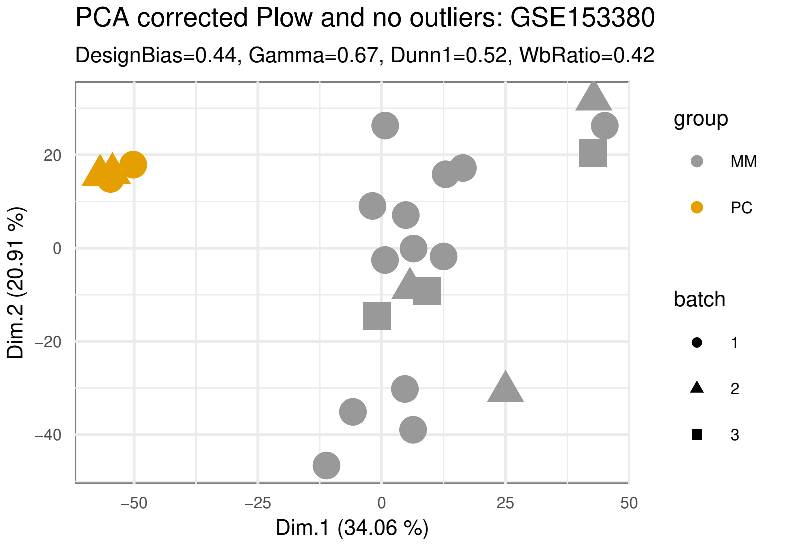
PCA corrected Plow and no outliers: GSE153380
DesignBias=0.44, Gamma=0.67, Dunn1=0.52, WbRatio=0.42
Dim.1 (34.06 %)
Dim.2 (20.91 %)
group
MM
PC
batch
1
2
3

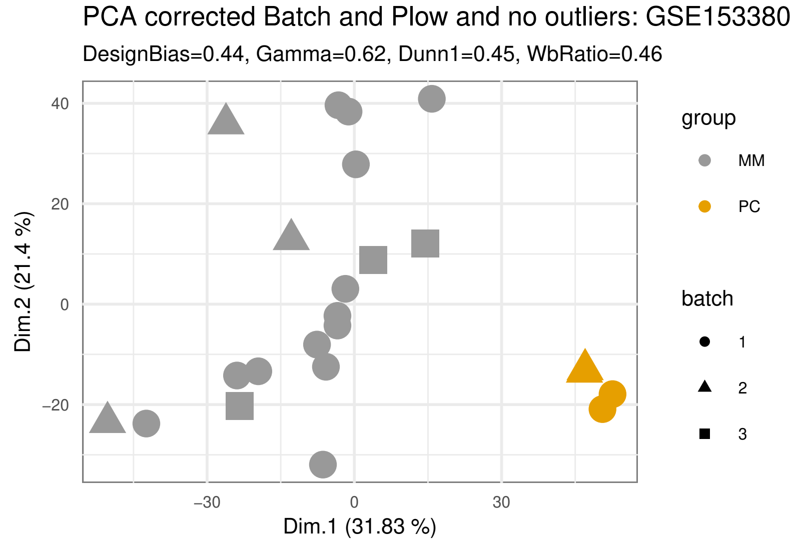
PCA corrected Batch and Plow and no outliers: GSE153380
DesignBias=0.44, Gamma=0.62, Dunn1=0.45, WbRatio=0.46
Dim.1 (31.83 %)
Dim.2 (21.4 %)
group
MM
PC
batch
1
2
3

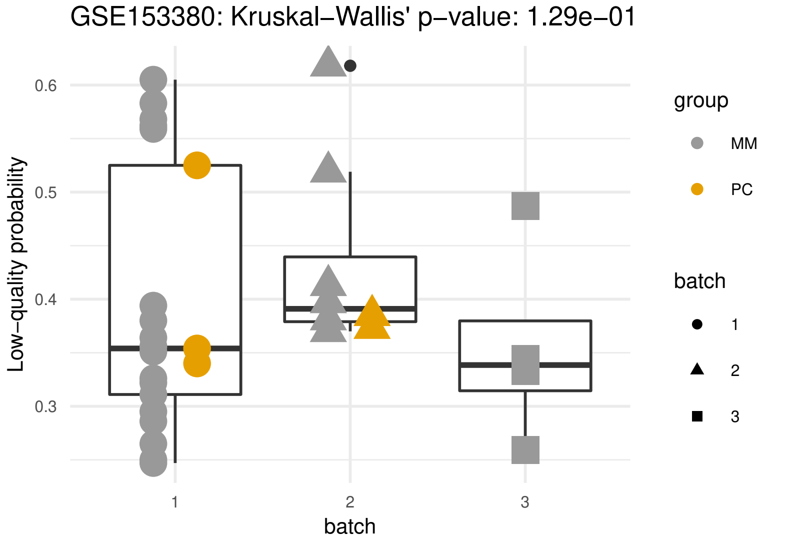
GSE153380: Kruskal−Wallis' p−value: 1.29e−01
Low−quality probability
batch
group
MM
PC
batch
1
2
3

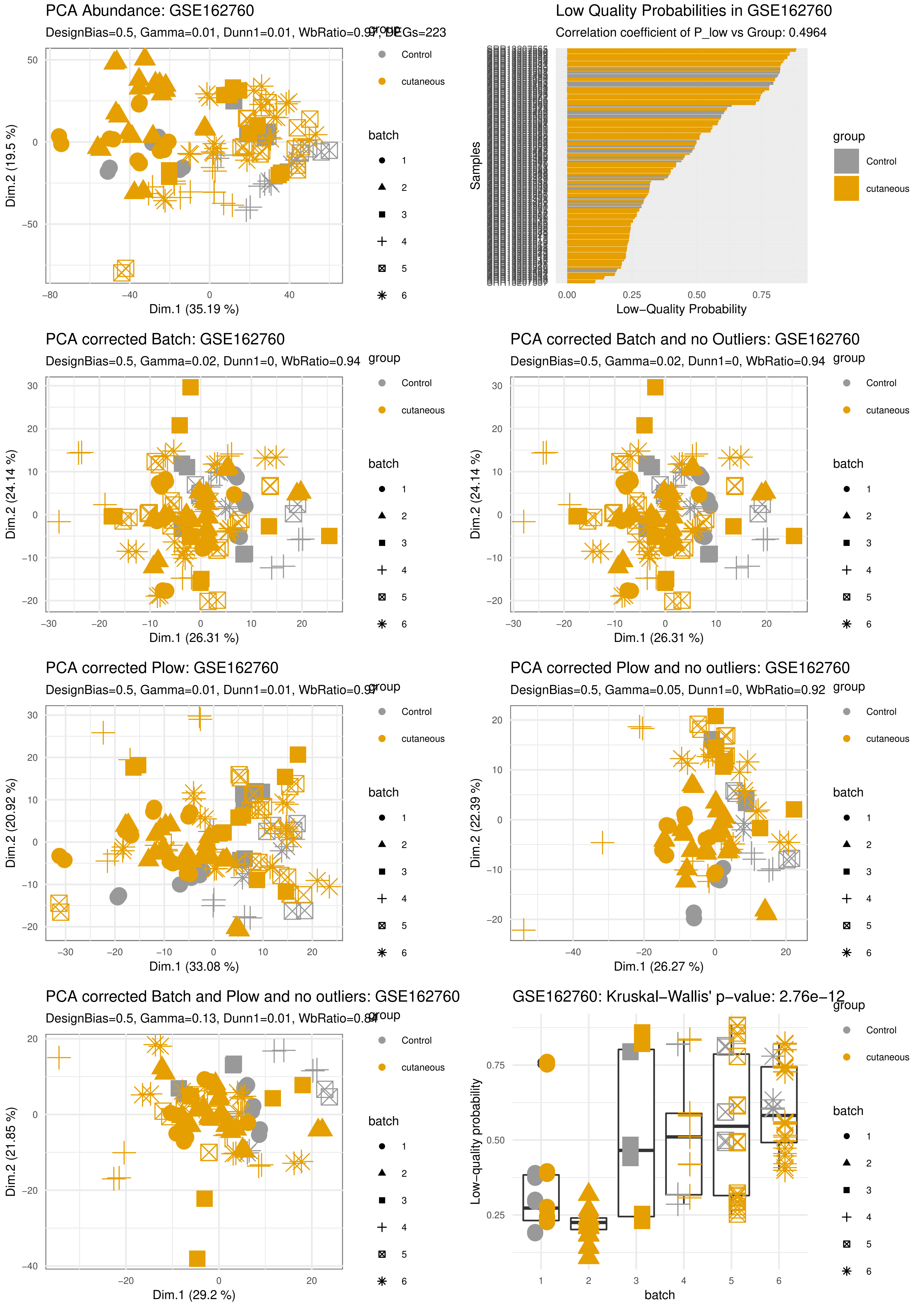
PCA Abundance: GSE162760
DesignBias=0.5, Gamma=0.01, Dunn1=0.01, WbRatio=0.97, DEGs=223
Dim.1 (35.19 %)
Dim.2 (19.5 %)
Low Quality Probabilities in GSE162760
Correlation coefficient of P_low vs Group: 0.4964
Samples
Low−Quality Probability
PCA corrected Batch: GSE162760
DesignBias=0.5, Gamma=0.02, Dunn1=0, WbRatio=0.94
Dim.1 (26.31 %)
Dim.2 (24.14 %)
PCA corrected Batch and no Outliers: GSE162760
DesignBias=0.5, Gamma=0.02, Dunn1=0, WbRatio=0.94
Dim.1 (26.31 %)
Dim.2 (24.14 %)
PCA corrected Plow: GSE162760
DesignBias=0.5, Gamma=0.01, Dunn1=0.01, WbRatio=0.97
Dim.1 (33.08 %)
Dim.2 (20.92 %)
PCA corrected Plow and no outliers: GSE162760
DesignBias=0.5, Gamma=0.05, Dunn1=0, WbRatio=0.92
Dim.1 (26.27 %)
Dim.2 (22.39 %)
PCA corrected Batch and Plow and no outliers: GSE162760
DesignBias=0.5, Gamma=0.13, Dunn1=0.01, WbRatio=0.84
Dim.1 (29.2 %)
Dim.2 (21.85 %)
GSE162760: Kruskal−Wallis' p−value: 2.76e−12
Low−quality probability
batch
group
Control
cutaneous
batch
1
2
3
4
5
6

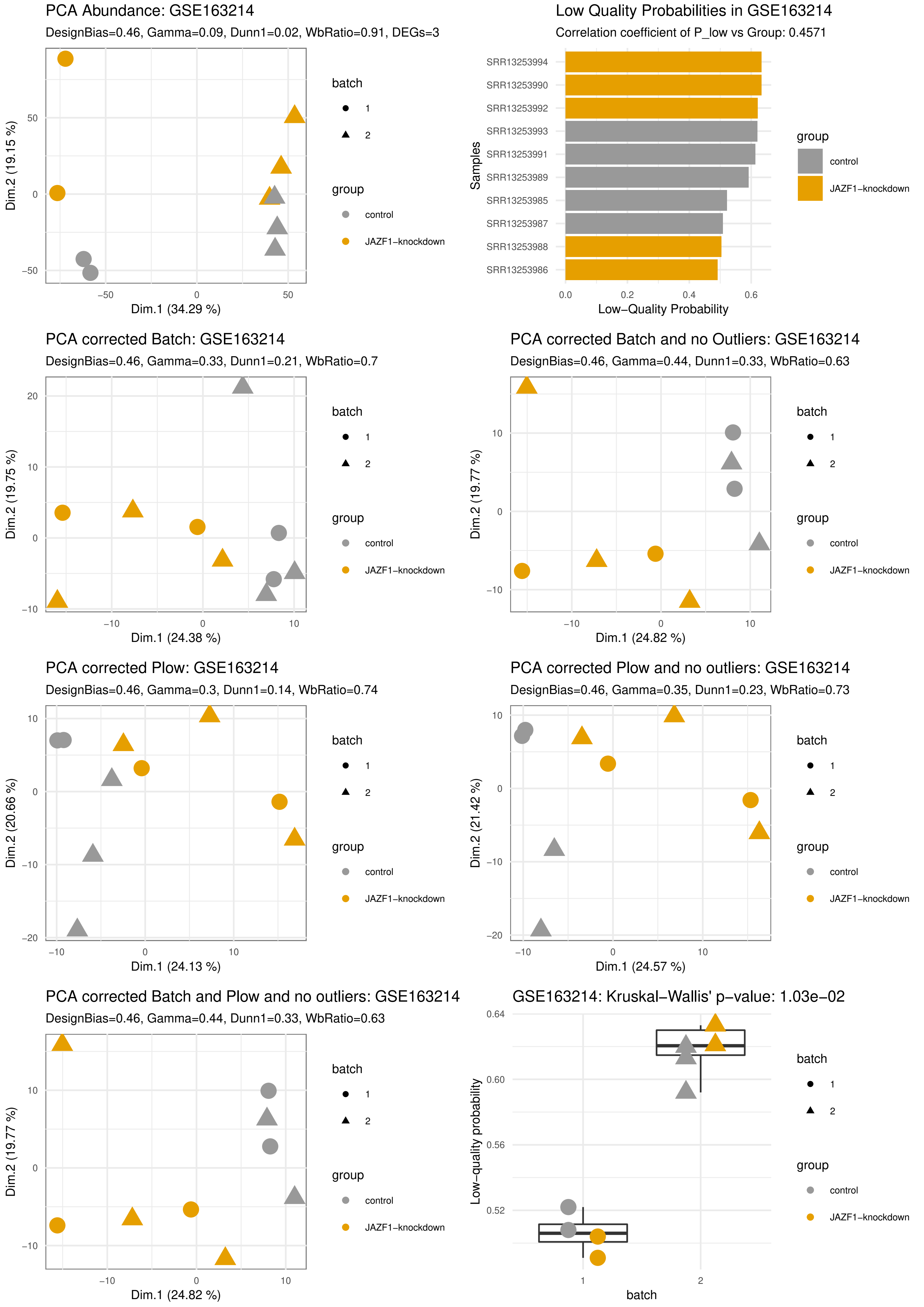
PCA Abundance: GSE163214
DesignBias=0.46, Gamma=0.09, Dunn1=0.02, WbRatio=0.91, DEGs=3
Dim.1 (34.29 %)
Dim.2 (19.15 %)
batch
1
2
group
control
JAZF1−knockdown
Low Quality Probabilities in GSE163214
Correlation coefficient of P_low vs Group: 0.4571
SRR13253994
SRR13253990
SRR13253992
SRR13253993
SRR13253991
SRR13253989
SRR13253985
SRR13253987
SRR13253988
SRR13253986
Samples
Low−Quality Probability
PCA corrected Batch: GSE163214
DesignBias=0.46, Gamma=0.33, Dunn1=0.21, WbRatio=0.7
Dim.1 (24.38 %)
Dim.2 (19.75 %)
PCA corrected Batch and no Outliers: GSE163214
DesignBias=0.46, Gamma=0.44, Dunn1=0.33, WbRatio=0.63
Dim.1 (24.82 %)
Dim.2 (19.77 %)
PCA corrected Plow: GSE163214
DesignBias=0.46, Gamma=0.3, Dunn1=0.14, WbRatio=0.74
Dim.1 (24.13 %)
Dim.2 (20.66 %)
PCA corrected Plow and no outliers: GSE163214
DesignBias=0.46, Gamma=0.35, Dunn1=0.23, WbRatio=0.73
Dim.1 (24.57 %)
Dim.2 (21.42 %)
PCA corrected Batch and Plow and no outliers: GSE163214
DesignBias=0.46, Gamma=0.44, Dunn1=0.33, WbRatio=0.63
Dim.1 (24.82 %)
Dim.2 (19.77 %)
GSE163214: Kruskal−Wallis' p-value: 1.03e−02
Low−quality probability
batch

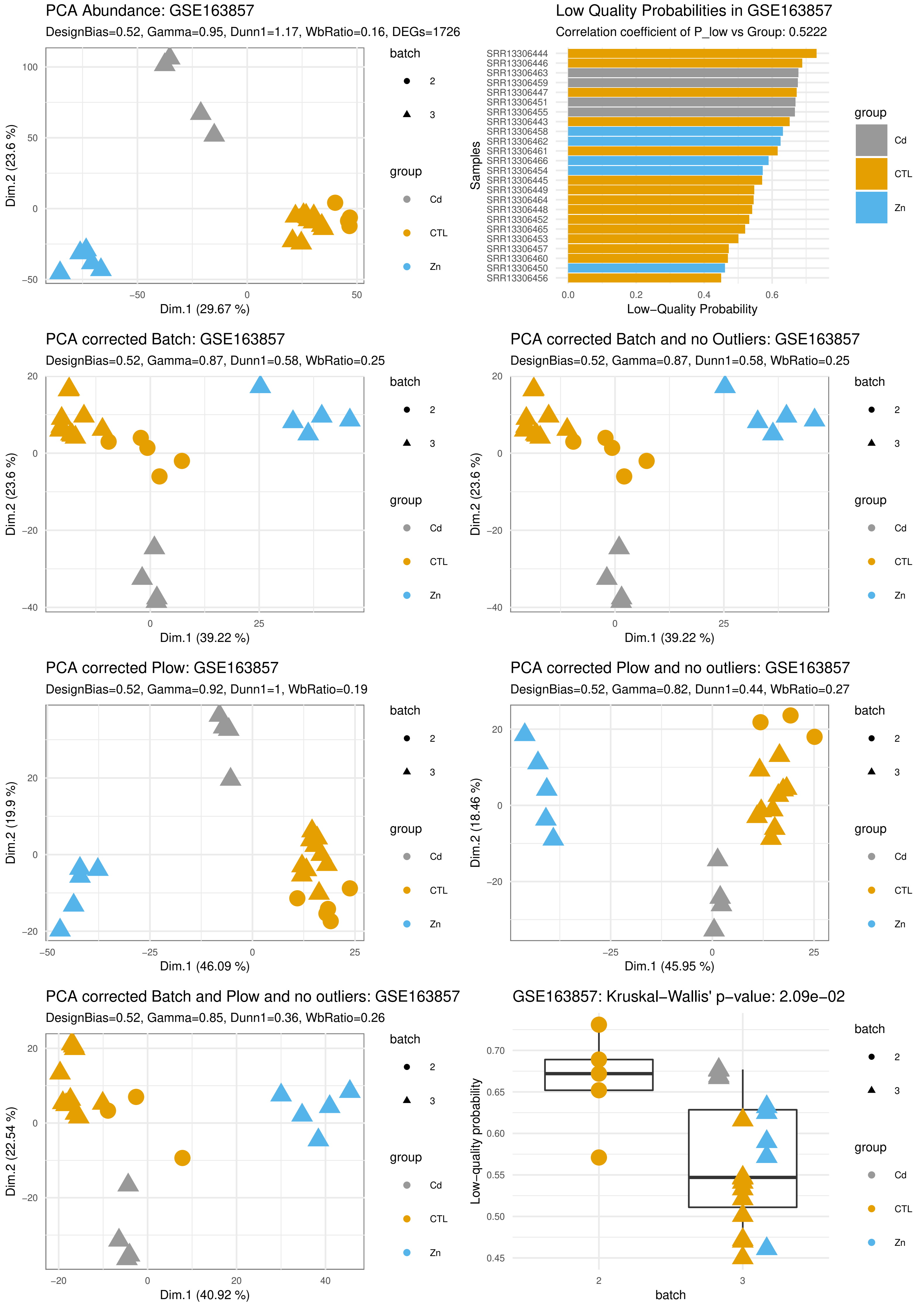
PCA Abundance: GSE163857
DesignBias=0.52, Gamma=0.95, Dunn1=1.17, WbRatio=0.16, DEGs=1726
Dim.1 (29.67 %)
Dim.2 (23.6 %)
batch
2
3
group
Cd
CTL
Zn
Low Quality Probabilities in GSE163857
Correlation coefficient of P_low vs Group: 0.5222
SRR13306444
SRR13306446
SRR13306463
SRR13306459
SRR13306447
SRR13306451
SRR13306455
SRR13306443
SRR13306458
SRR13306462
SRR13306461
SRR13306466
SRR13306454
SRR13306445
SRR13306449
SRR13306464
SRR13306448
SRR13306452
SRR13306465
SRR13306453
SRR13306457
SRR13306460
SRR13306450
SRR13306456
Samples
Low−Quality Probability
group
Cd
CTL
Zn
PCA corrected Batch: GSE163857
DesignBias=0.52, Gamma=0.87, Dunn1=0.58, WbRatio=0.25
Dim.1 (39.22 %)
Dim.2 (23.6 %)
PCA corrected Batch and no Outliers: GSE163857
DesignBias=0.52, Gamma=0.87, Dunn1=0.58, WbRatio=0.25
Dim.1 (39.22 %)
Dim.2 (23.6 %)
PCA corrected Plow: GSE163857
DesignBias=0.52, Gamma=0.92, Dunn1=1, WbRatio=0.19
Dim.1 (46.09 %)
Dim.2 (19.9 %)
PCA corrected Plow and no outliers: GSE163857
DesignBias=0.52, Gamma=0.82, Dunn1=0.44, WbRatio=0.27
Dim.1 (45.95 %)
Dim.2 (18.46 %)
PCA corrected Batch and Plow and no outliers: GSE163857
DesignBias=0.52, Gamma=0.85, Dunn1=0.36, WbRatio=0.26
Dim.1 (40.92 %)
Dim.2 (22.54 %)
GSE163857: Kruskal−Wallis' p−value: 2.09e−02
Low−quality probability
batch

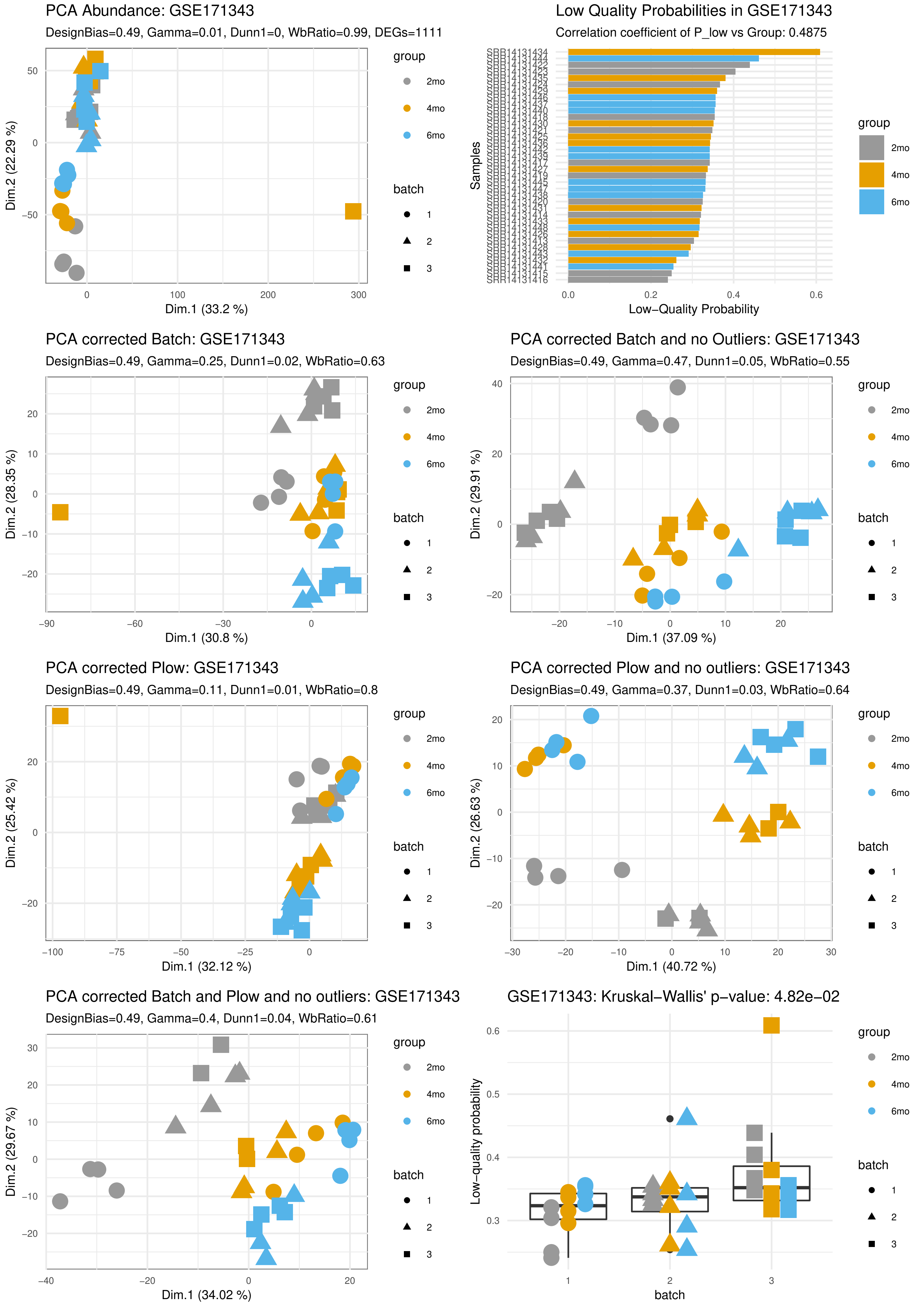
PCA Abundance: GSE171343
DesignBias=0.49, Gamma=0.01, Dunn1=0, WbRatio=0.99, DEGs=1111
Dim.1 (33.2 %)
Dim.2 (22.29 %)
Low Quality Probabilities in GSE171343
Correlation coefficient of P_low vs Group: 0.4875
Samples
Low−Quality Probability
PCA corrected Batch: GSE171343
DesignBias=0.49, Gamma=0.25, Dunn1=0.02, WbRatio=0.63
Dim.1 (30.8 %)
Dim.2 (28.35 %)
PCA corrected Batch and no Outliers: GSE171343
DesignBias=0.49, Gamma=0.47, Dunn1=0.05, WbRatio=0.55
Dim.1 (37.09 %)
Dim.2 (29.91 %)
PCA corrected Plow: GSE171343
DesignBias=0.49, Gamma=0.11, Dunn1=0.01, WbRatio=0.8
Dim.1 (32.12 %)
Dim.2 (25.42 %)
PCA corrected Plow and no outliers: GSE171343
DesignBias=0.49, Gamma=0.37, Dunn1=0.03, WbRatio=0.64
Dim.1 (40.72 %)
Dim.2 (26.63 %)
PCA corrected Batch and Plow and no outliers: GSE171343
DesignBias=0.49, Gamma=0.4, Dunn1=0.04, WbRatio=0.61
Dim.1 (34.02 %)
Dim.2 (29.67 %)
GSE171343: Kruskal−Wallis' p-value: 4.82e−02
Low−quality probability
batch
group
2mo
4mo
6mo
batch
1
2
3

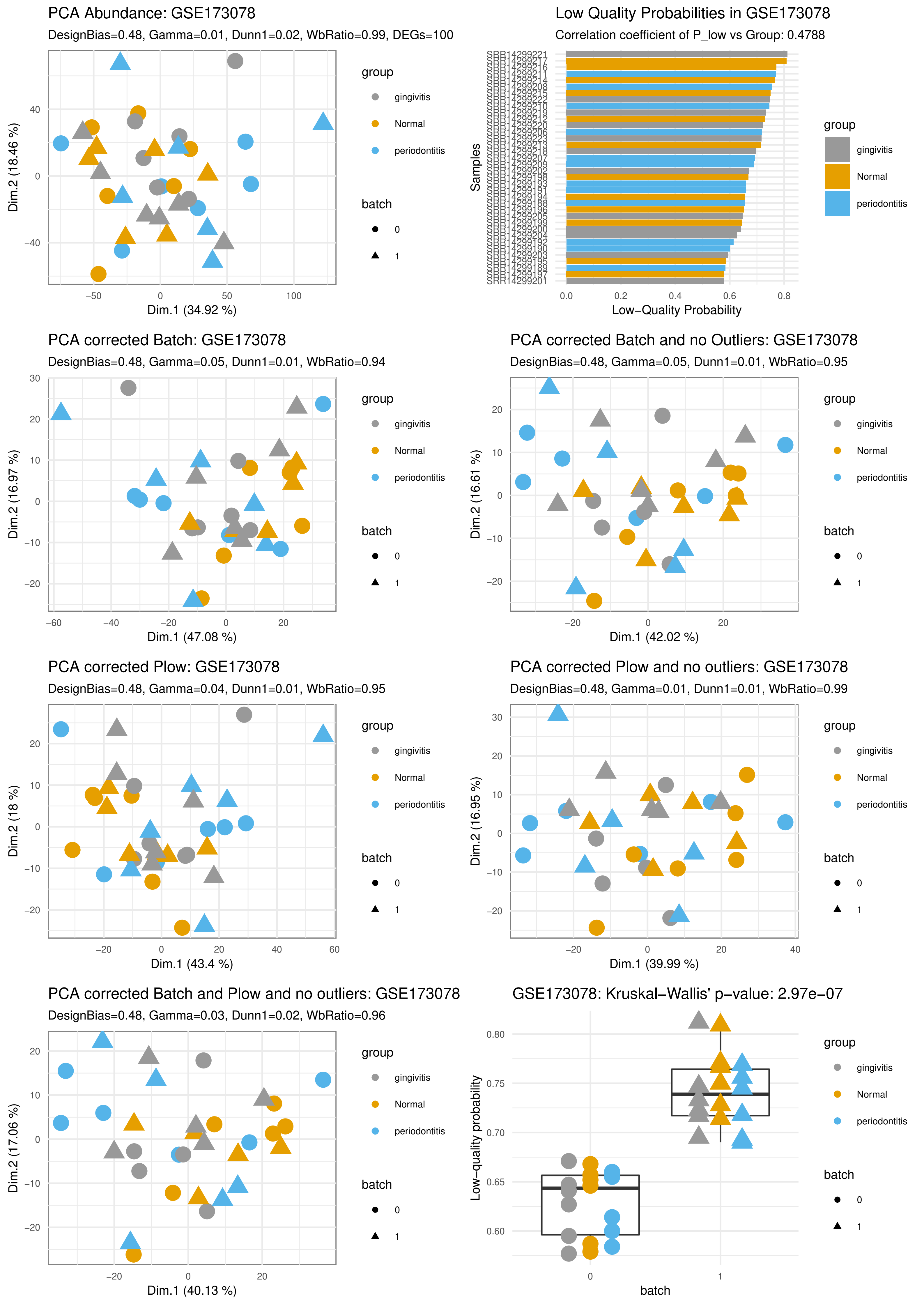

PCA Abundance: GSE173078
DesignBias=0.48, Gamma=0.01, Dunn1=0.02, WbRatio=0.99, DEGs=100
Dim.1 (34.92 %)
Dim.2 (18.46 %)
group
gingivitis
Normal
periodontitis
batch
0
1
Low Quality Probabilities in GSE173078
Correlation coefficient of P_low vs Group: 0.4788
Samples
Low−Quality Probability
PCA corrected Batch: GSE173078
DesignBias=0.48, Gamma=0.05, Dunn1=0.01, WbRatio=0.94
Dim.1 (47.08 %)
Dim.2 (16.97 %)
PCA corrected Batch and no Outliers: GSE173078
DesignBias=0.48, Gamma=0.05, Dunn1=0.01, WbRatio=0.95
Dim.1 (42.02 %)
Dim.2 (16.61 %)
PCA corrected Plow: GSE173078
DesignBias=0.48, Gamma=0.04, Dunn1=0.01, WbRatio=0.95
Dim.1 (43.4 %)
Dim.2 (18 %)
PCA corrected Plow and no outliers: GSE173078
DesignBias=0.48, Gamma=0.01, Dunn1=0.01, WbRatio=0.99
Dim.1 (39.99 %)
Dim.2 (16.95 %)
PCA corrected Batch and Plow and no outliers: GSE173078
DesignBias=0.48, Gamma=0.03, Dunn1=0.02, WbRatio=0.96
Dim.1 (40.13 %)
Dim.2 (17.06 %)
GSE173078: Kruskal−Wallis' p−value: 2.97e−07
Low−quality probability
batch

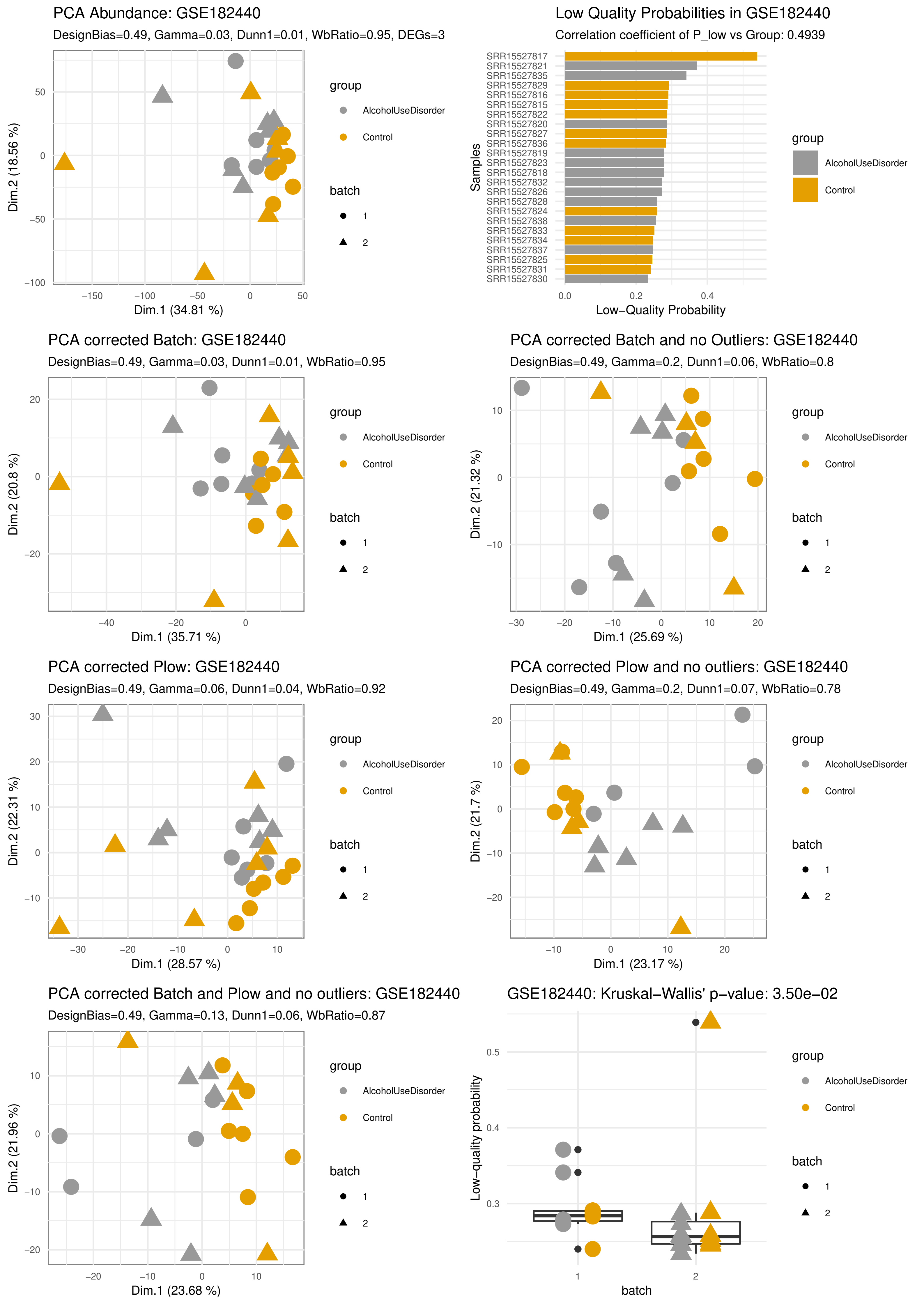

PCA Abundance: GSE182440
DesignBias=0.49, Gamma=0.03, Dunn1=0.01, WbRatio=0.95, DEGs=3
Dim.1 (34.81 %)
Dim.2 (18.56 %)
group
AlcoholUseDisorder
Control
batch
1
2
Low Quality Probabilities in GSE182440
Correlation coefficient of P_low vs Group: 0.4939
Samples
SRR15527817
SRR15527821
SRR15527835
SRR15527829
SRR15527816
SRR15527815
SRR15527822
SRR15527820
SRR15527827
SRR15527836
SRR15527819
SRR15527823
SRR15527818
SRR15527832
SRR15527826
SRR15527828
SRR15527824
SRR15527838
SRR15527833
SRR15527834
SRR15527837
SRR15527825
SRR15527831
SRR15527830
Low−Quality Probability
PCA corrected Batch: GSE182440
DesignBias=0.49, Gamma=0.03, Dunn1=0.01, WbRatio=0.95
Dim.1 (35.71 %)
Dim.2 (20.8 %)
PCA corrected Batch and no Outliers: GSE182440
DesignBias=0.49, Gamma=0.2, Dunn1=0.06, WbRatio=0.8
Dim.1 (25.69 %)
Dim.2 (21.32 %)
PCA corrected Plow: GSE182440
DesignBias=0.49, Gamma=0.06, Dunn1=0.04, WbRatio=0.92
Dim.1 (28.57 %)
Dim.2 (22.31 %)
PCA corrected Plow and no outliers: GSE182440
DesignBias=0.49, Gamma=0.2, Dunn1=0.07, WbRatio=0.78
Dim.1 (23.17 %)
Dim.2 (21.7 %)
PCA corrected Batch and Plow and no outliers: GSE182440
DesignBias=0.49, Gamma=0.13, Dunn1=0.06, WbRatio=0.87
Dim.1 (23.68 %)
Dim.2 (21.96 %)
GSE182440: Kruskal−Wallis' p-value: 3.50e−02
Low−quality probability
batch
