# Supplementary material for: Batch effect detection and correction in RNA-seq data using machine-learning-based automated assessment of quality
Source: BMC Bioinformatics. 2022 Jul 14;23(Suppl 6):279. doi: 10.1186/s12859-022-04775-y (PMC9284682; doi:10.1186/s12859-022-04775-y)
Supplement: Supplementary file 2 — Additional file 2. Comparison of Plow and SVA Correction. [file 12859_2022_4775_MOESM2_ESM.pdf]

Supplementary material S2

From top left to bottom right: PCA Abundance, shows the uncorrected PCA of the rlog normalized counts quantified by salmon and imported to a deseq2 object, next to it in the top right panel: The PCA corrected with batch using the acPCA package to return principal components that were computed with the true batch as a confounding factor. The PCA corrected with P_{low} uses the acPCA package likewise, but P_{low} as a confounding factor. To the right of the P_{low} corrected PCA is a corrected PCA on the basis of the data without outliers. We removed outliers of base or corrected PCA, but additionally added a threshold for P_{low} depending on the distribution of P_{low} in the samples (See S1). The last two panels are the PCA corrected by the first surrogate variable of the sva-method and the PCA corrected with all surrogate variables found for this dataset.

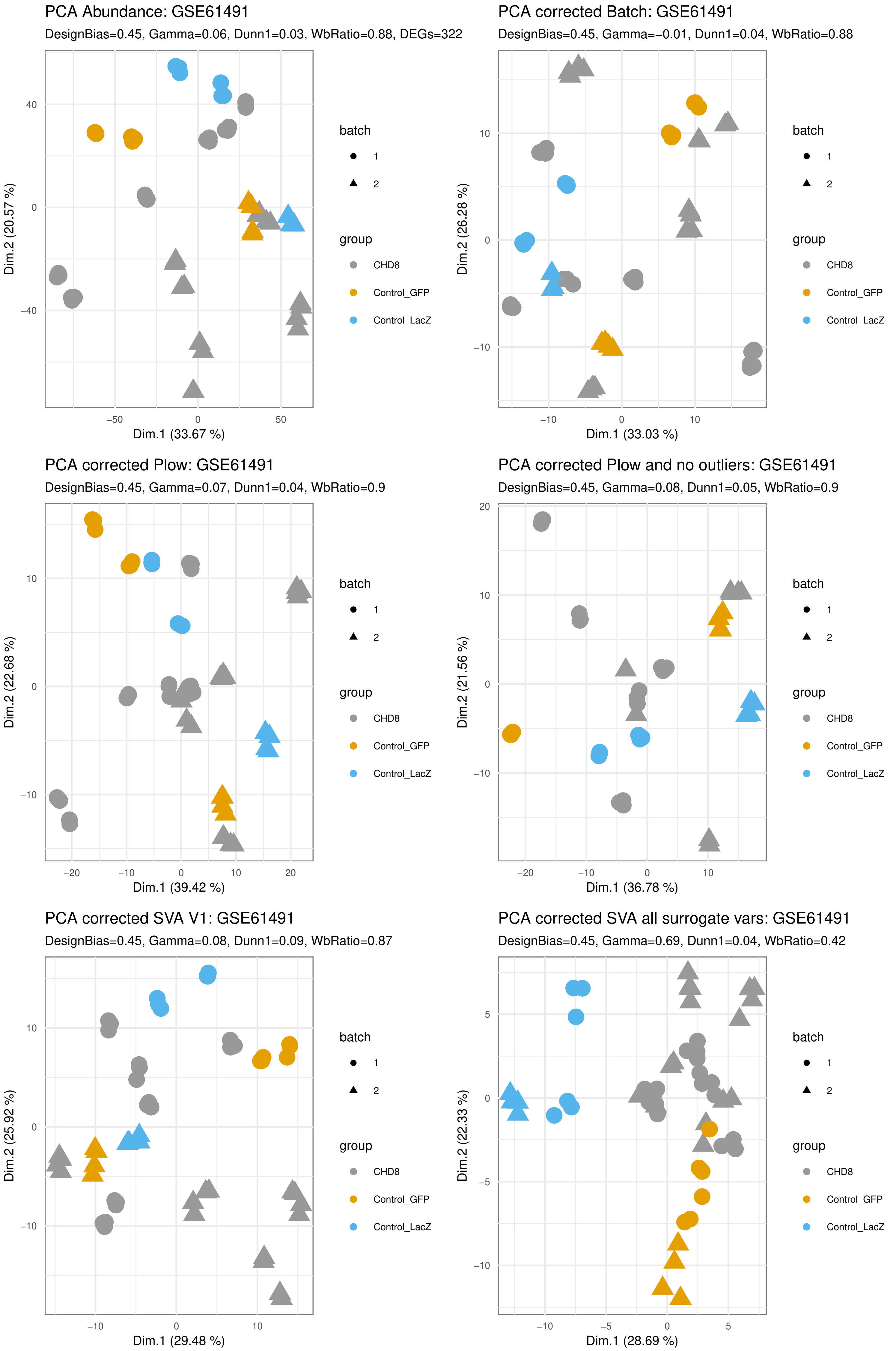

PCA Abundance: GSE61491
DesignBias=0.45, Gamma=0.06, Dunn1=0.03, WbRatio=0.88, DEGs=322
Dim.1 (33.67 %)
Dim.2 (20.57 %)
PCA corrected Batch: GSE61491
DesignBias=0.45, Gamma=−0.01, Dunn1=0.04, WbRatio=0.88
Dim.1 (33.03 %)
Dim.2 (26.28 %)
PCA corrected Plow: GSE61491
DesignBias=0.45, Gamma=0.07, Dunn1=0.04, WbRatio=0.9
Dim.1 (39.42 %)
Dim.2 (22.68 %)
PCA corrected Plow and no outliers: GSE61491
DesignBias=0.45, Gamma=0.08, Dunn1=0.05, WbRatio=0.9
Dim.1 (36.78 %)
Dim.2 (21.56 %)
PCA corrected SVA V1: GSE61491
DesignBias=0.45, Gamma=0.08, Dunn1=0.09, WbRatio=0.87
Dim.1 (29.48 %)
Dim.2 (25.92 %)
PCA corrected SVA all surrogate vars: GSE61491
DesignBias=0.45, Gamma=0.69, Dunn1=0.04, WbRatio=0.42
Dim.1 (28.69 %)
Dim.2 (22.33 %)
batch
1
2
group
CHD8
Control_GFP
Control_LacZ

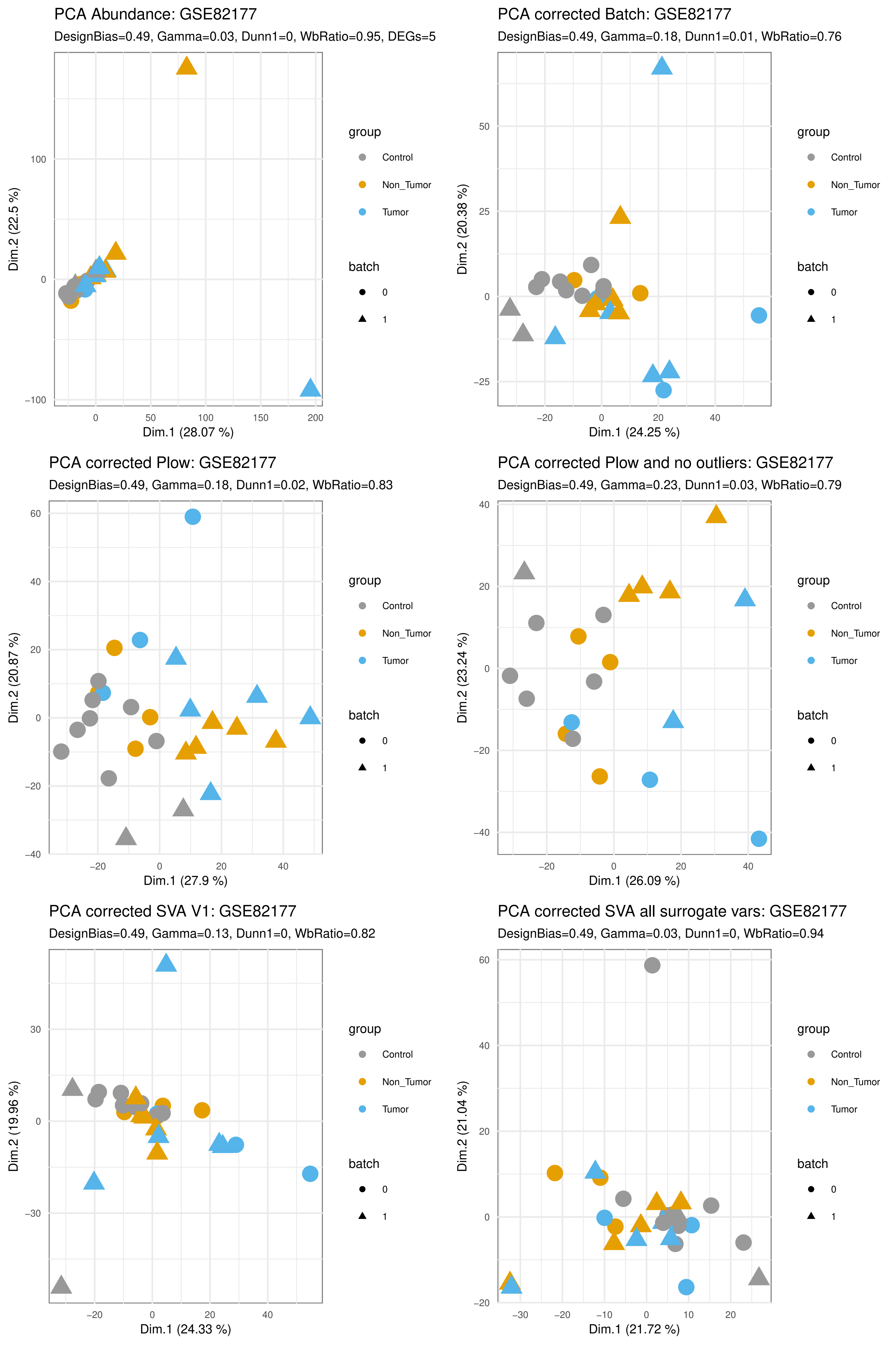
PCA Abundance: GSE82177
DesignBias=0.49, Gamma=0.03, Dunn1=0, WbRatio=0.95, DEGs=5
Dim.1 (28.07 %)
Dim.2 (22.5 %)
PCA corrected Batch: GSE82177
DesignBias=0.49, Gamma=0.18, Dunn1=0.01, WbRatio=0.76
Dim.1 (24.25 %)
Dim.2 (20.38 %)
PCA corrected Plow: GSE82177
DesignBias=0.49, Gamma=0.18, Dunn1=0.02, WbRatio=0.83
Dim.1 (27.9 %)
Dim.2 (20.87 %)
PCA corrected Plow and no outliers: GSE82177
DesignBias=0.49, Gamma=0.23, Dunn1=0.03, WbRatio=0.79
Dim.1 (26.09 %)
Dim.2 (23.24 %)
PCA corrected SVA V1: GSE82177
DesignBias=0.49, Gamma=0.13, Dunn1=0, WbRatio=0.82
Dim.1 (24.33 %)
Dim.2 (19.96 %)
PCA corrected SVA all surrogate vars: GSE82177
DesignBias=0.49, Gamma=0.03, Dunn1=0, WbRatio=0.94
Dim.1 (21.72 %)
Dim.2 (21.04 %)
group
Control
Non_Tumor
Tumor
batch
0
1

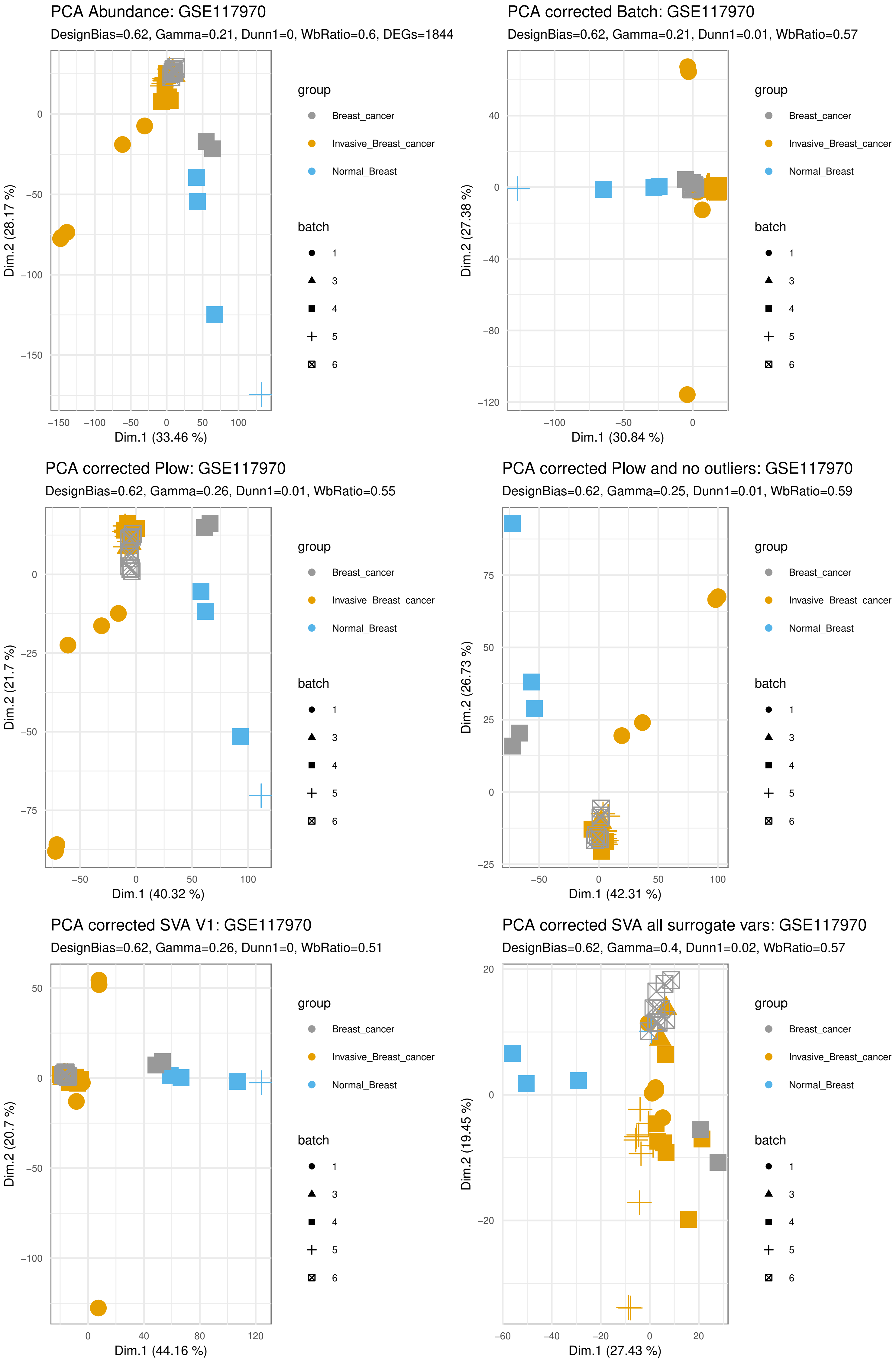
PCA Abundance: GSE117970
DesignBias=0.62, Gamma=0.21, Dunn1=0, WbRatio=0.6, DEGs=1844
Dim.1 (33.46 %)
Dim.2 (28.17 %)
PCA corrected Batch: GSE117970
DesignBias=0.62, Gamma=0.21, Dunn1=0.01, WbRatio=0.57
Dim.1 (30.84 %)
Dim.2 (27.38 %)
PCA corrected Plow: GSE117970
DesignBias=0.62, Gamma=0.26, Dunn1=0.01, WbRatio=0.55
Dim.1 (40.32 %)
Dim.2 (21.7 %)
PCA corrected Plow and no outliers: GSE117970
DesignBias=0.62, Gamma=0.25, Dunn1=0.01, WbRatio=0.59
Dim.1 (42.31 %)
Dim.2 (26.73 %)
PCA corrected SVA V1: GSE117970
DesignBias=0.62, Gamma=0.26, Dunn1=0, WbRatio=0.51
Dim.1 (44.16 %)
Dim.2 (20.7 %)
PCA corrected SVA all surrogate vars: GSE117970
DesignBias=0.62, Gamma=0.4, Dunn1=0.02, WbRatio=0.57
Dim.1 (27.43 %)
Dim.2 (19.45 %)
group
Breast_cancer
Invasive_Breast_cancer
Normal_Breast
batch
1
3
4
5
6

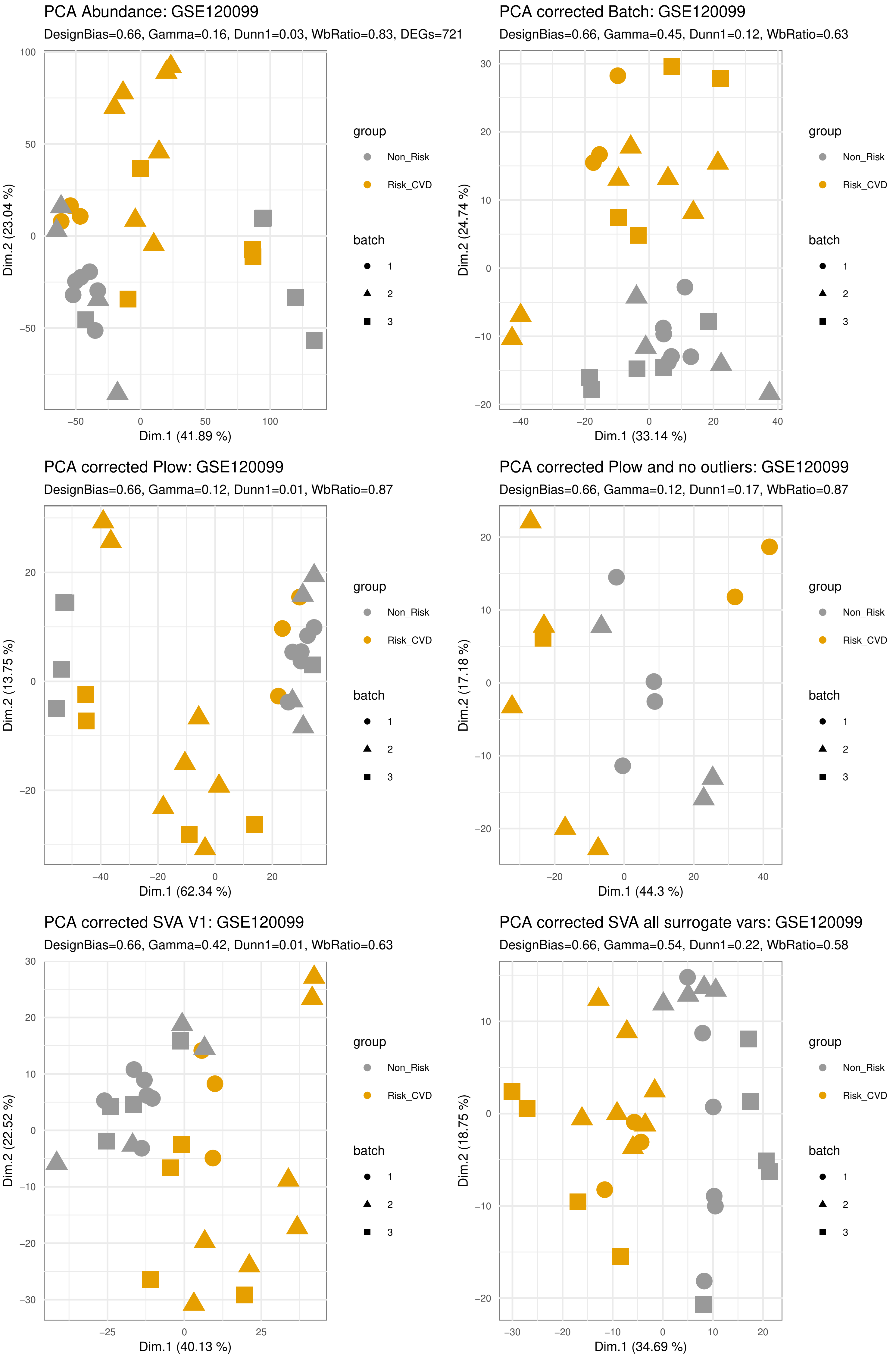
PCA Abundance: GSE120099
DesignBias=0.66, Gamma=0.16, Dunn1=0.03, WbRatio=0.83, DEGs=721
Dim.1 (41.89 %)
Dim.2 (23.04 %)
PCA corrected Batch: GSE120099
DesignBias=0.66, Gamma=0.45, Dunn1=0.12, WbRatio=0.63
Dim.1 (33.14 %)
Dim.2 (24.74 %)
PCA corrected Plow: GSE120099
DesignBias=0.66, Gamma=0.12, Dunn1=0.01, WbRatio=0.87
Dim.1 (62.34 %)
Dim.2 (13.75 %)
PCA corrected Plow and no outliers: GSE120099
DesignBias=0.66, Gamma=0.12, Dunn1=0.17, WbRatio=0.87
Dim.1 (44.3 %)
Dim.2 (17.18 %)
PCA corrected SVA V1: GSE120099
DesignBias=0.66, Gamma=0.42, Dunn1=0.01, WbRatio=0.63
Dim.1 (40.13 %)
Dim.2 (22.52 %)
PCA corrected SVA all surrogate vars: GSE120099
DesignBias=0.66, Gamma=0.54, Dunn1=0.22, WbRatio=0.58
Dim.1 (34.69 %)
Dim.2 (18.75 %)
group
Non_Risk
Risk_CVD
batch
1
2
3

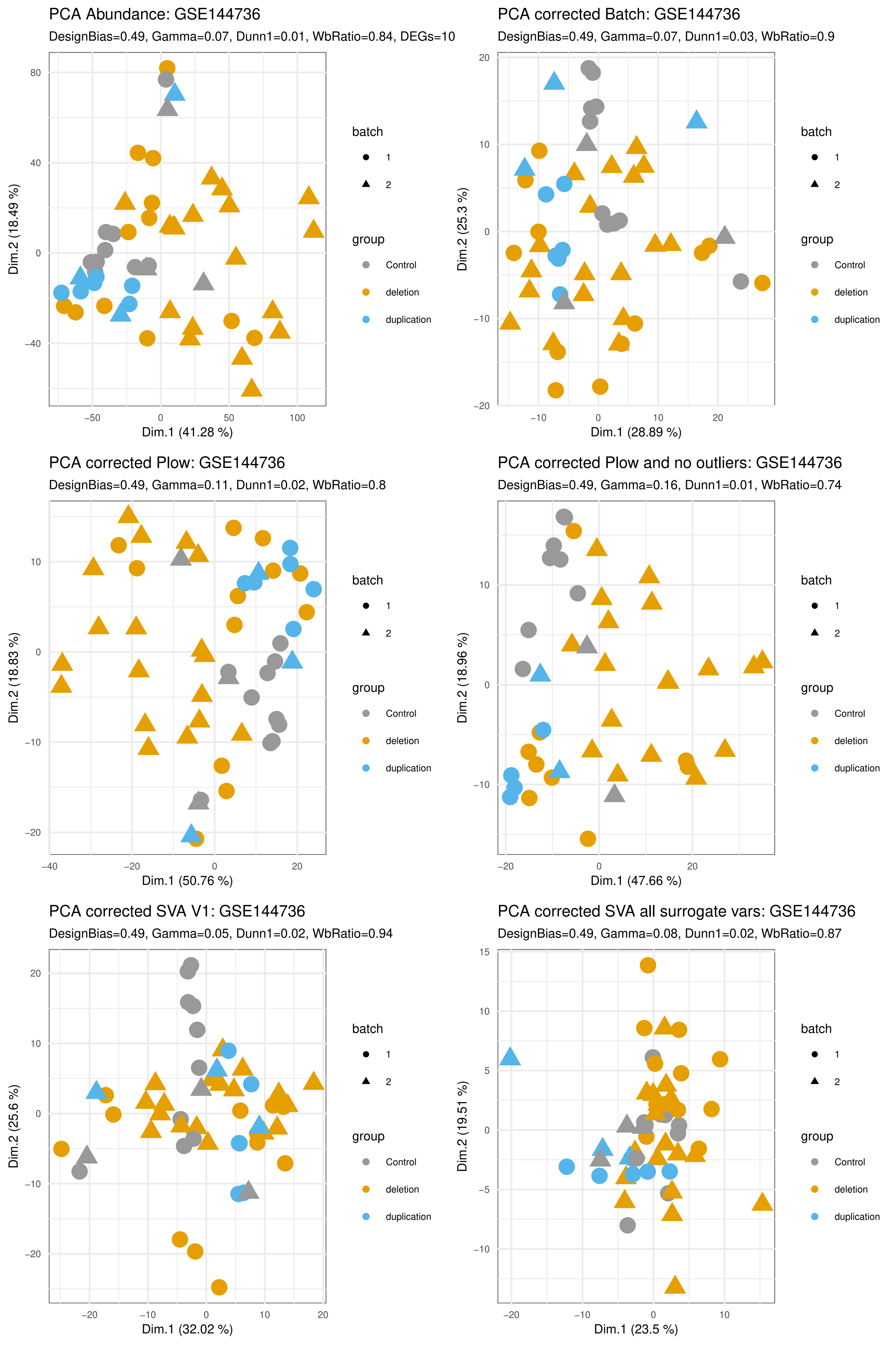

PCA Abundance: GSE144736
DesignBias=0.49, Gamma=0.07, Dunn1=0.01, WbRatio=0.84, DEGs=10
Dim.1 (41.28 %)
Dim.2 (18.49 %)
PCA corrected Batch: GSE144736
DesignBias=0.49, Gamma=0.07, Dunn1=0.03, WbRatio=0.9
Dim.1 (28.89 %)
Dim.2 (25.3 %)
PCA corrected Plow: GSE144736
DesignBias=0.49, Gamma=0.11, Dunn1=0.02, WbRatio=0.8
Dim.1 (50.76 %)
Dim.2 (18.83 %)
PCA corrected Plow and no outliers: GSE144736
DesignBias=0.49, Gamma=0.16, Dunn1=0.01, WbRatio=0.74
Dim.1 (47.66 %)
Dim.2 (18.96 %)
PCA corrected SVA V1: GSE144736
DesignBias=0.49, Gamma=0.05, Dunn1=0.02, WbRatio=0.94
Dim.1 (32.02 %)
Dim.2 (25.6 %)
PCA corrected SVA all surrogate vars: GSE144736
DesignBias=0.49, Gamma=0.08, Dunn1=0.02, WbRatio=0.87
Dim.1 (23.5 %)
Dim.2 (19.51 %)
batch
1
2
group
Control
deletion
duplication

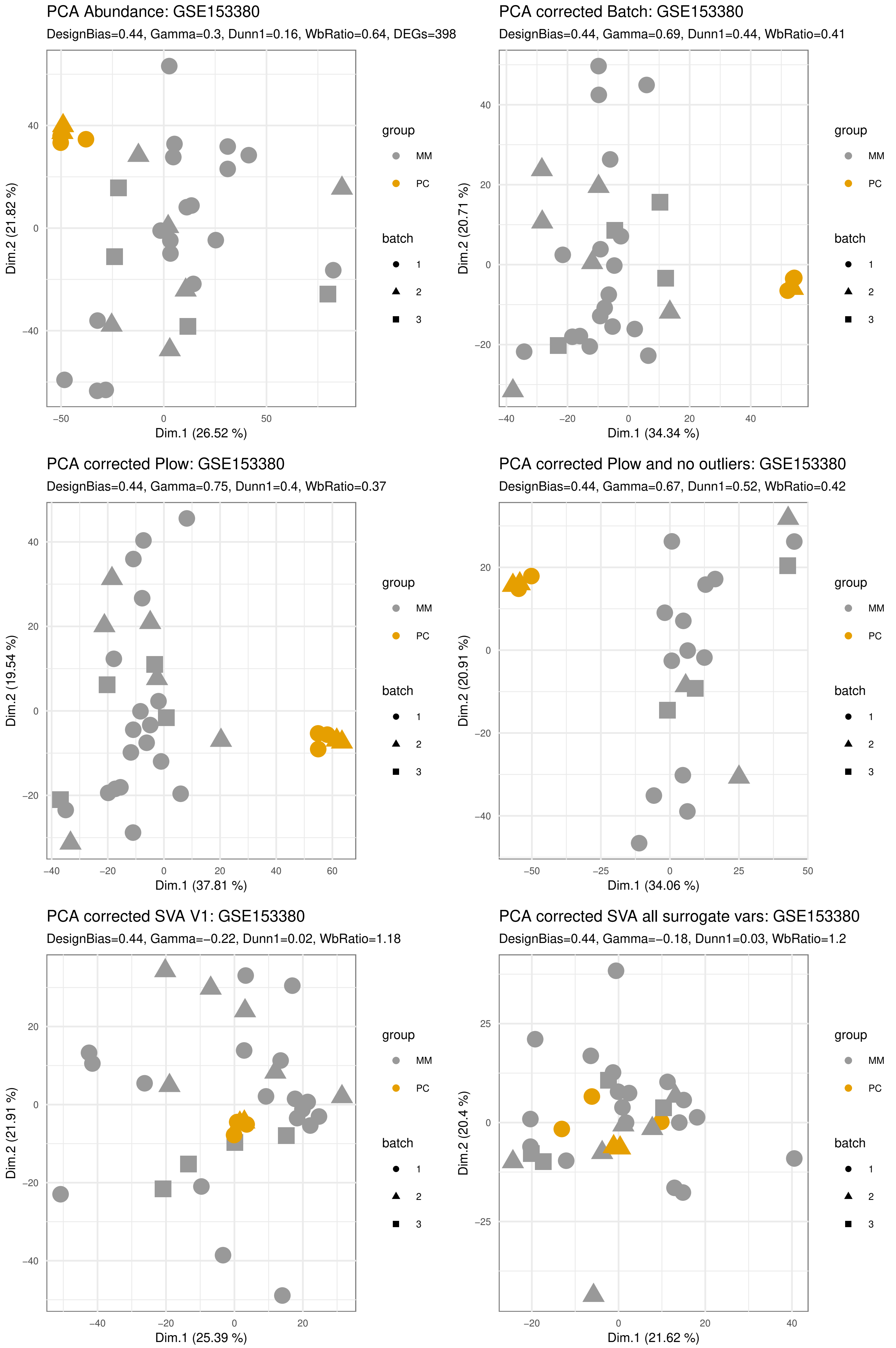
PCA Abundance: GSE153380
DesignBias=0.44, Gamma=0.3, Dunn1=0.16, WbRatio=0.64, DEGs=398
Dim.1 (26.52 %)
Dim.2 (21.82 %)
PCA corrected Batch: GSE153380
DesignBias=0.44, Gamma=0.69, Dunn1=0.44, WbRatio=0.41
Dim.1 (34.34 %)
Dim.2 (20.71 %)
PCA corrected Plow: GSE153380
DesignBias=0.44, Gamma=0.75, Dunn1=0.4, WbRatio=0.37
Dim.1 (37.81 %)
Dim.2 (19.54 %)
PCA corrected Plow and no outliers: GSE153380
DesignBias=0.44, Gamma=0.67, Dunn1=0.52, WbRatio=0.42
Dim.1 (34.06 %)
Dim.2 (20.91 %)
PCA corrected SVA V1: GSE153380
DesignBias=0.44, Gamma=−0.22, Dunn1=0.02, WbRatio=1.18
Dim.1 (25.39 %)
Dim.2 (21.91 %)
PCA corrected SVA all surrogate vars: GSE153380
DesignBias=0.44, Gamma=−0.18, Dunn1=0.03, WbRatio=1.2
Dim.1 (21.62 %)
Dim.2 (20.4 %)
group
MM
PC
batch
1
2
3

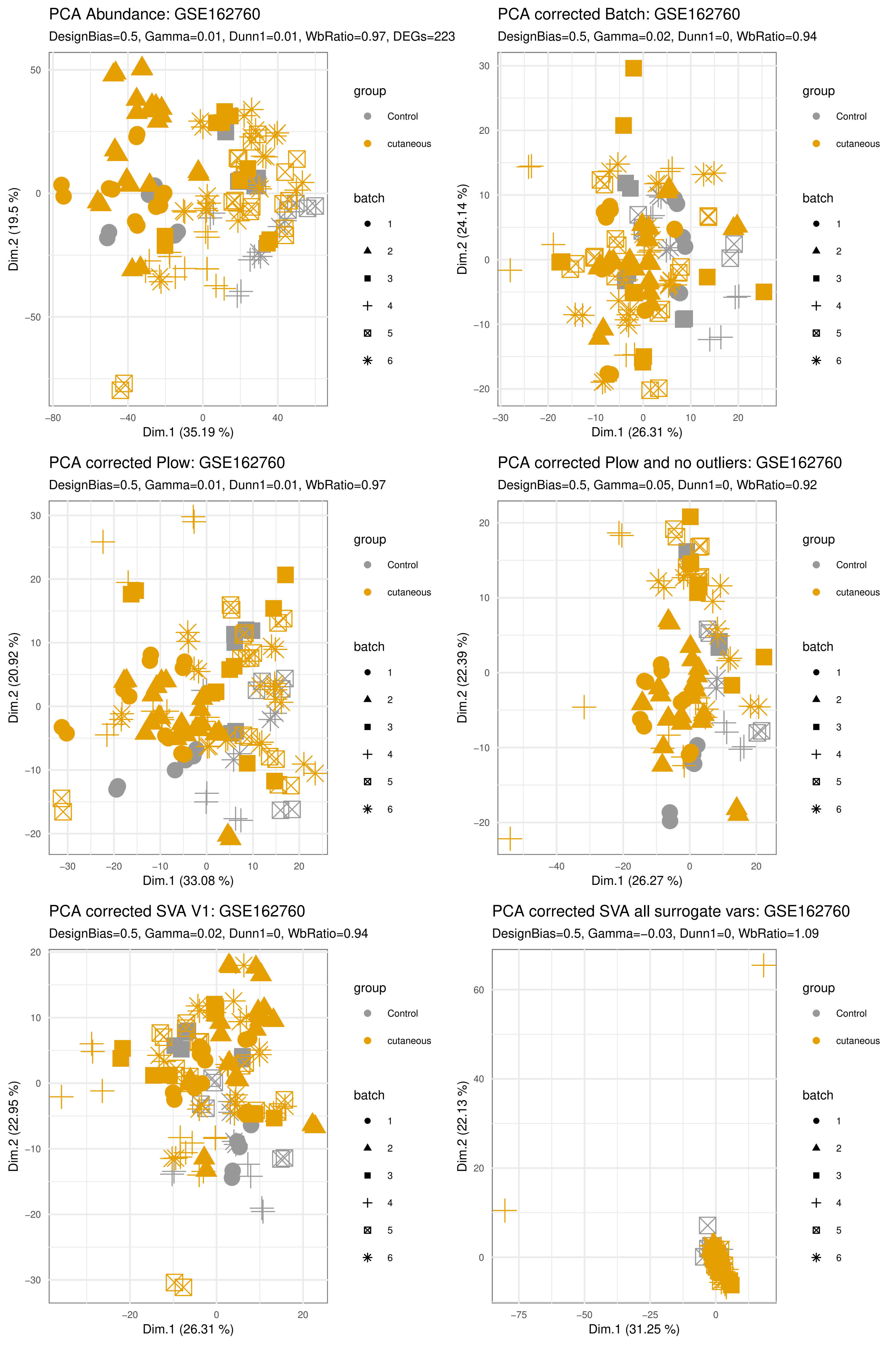
PCA Abundance: GSE162760
DesignBias=0.5, Gamma=0.01, Dunn1=0.01, WbRatio=0.97, DEGs=223
Dim.1 (35.19 %)
Dim.2 (19.5 %)
PCA corrected Batch: GSE162760
DesignBias=0.5, Gamma=0.02, Dunn1=0, WbRatio=0.94
Dim.1 (26.31 %)
Dim.2 (24.14 %)
PCA corrected Plow: GSE162760
DesignBias=0.5, Gamma=0.01, Dunn1=0.01, WbRatio=0.97
Dim.1 (33.08 %)
Dim.2 (20.92 %)
PCA corrected Plow and no outliers: GSE162760
DesignBias=0.5, Gamma=0.05, Dunn1=0, WbRatio=0.92
Dim.1 (26.27 %)
Dim.2 (22.39 %)
PCA corrected SVA V1: GSE162760
DesignBias=0.5, Gamma=0.02, Dunn1=0, WbRatio=0.94
Dim.1 (26.31 %)
Dim.2 (22.95 %)
PCA corrected SVA all surrogate vars: GSE162760
DesignBias=0.5, Gamma=−0.03, Dunn1=0, WbRatio=1.09
Dim.1 (31.25 %)
Dim.2 (22.13 %)
group
Control
cutaneous
batch
1
2
3
4
5
6

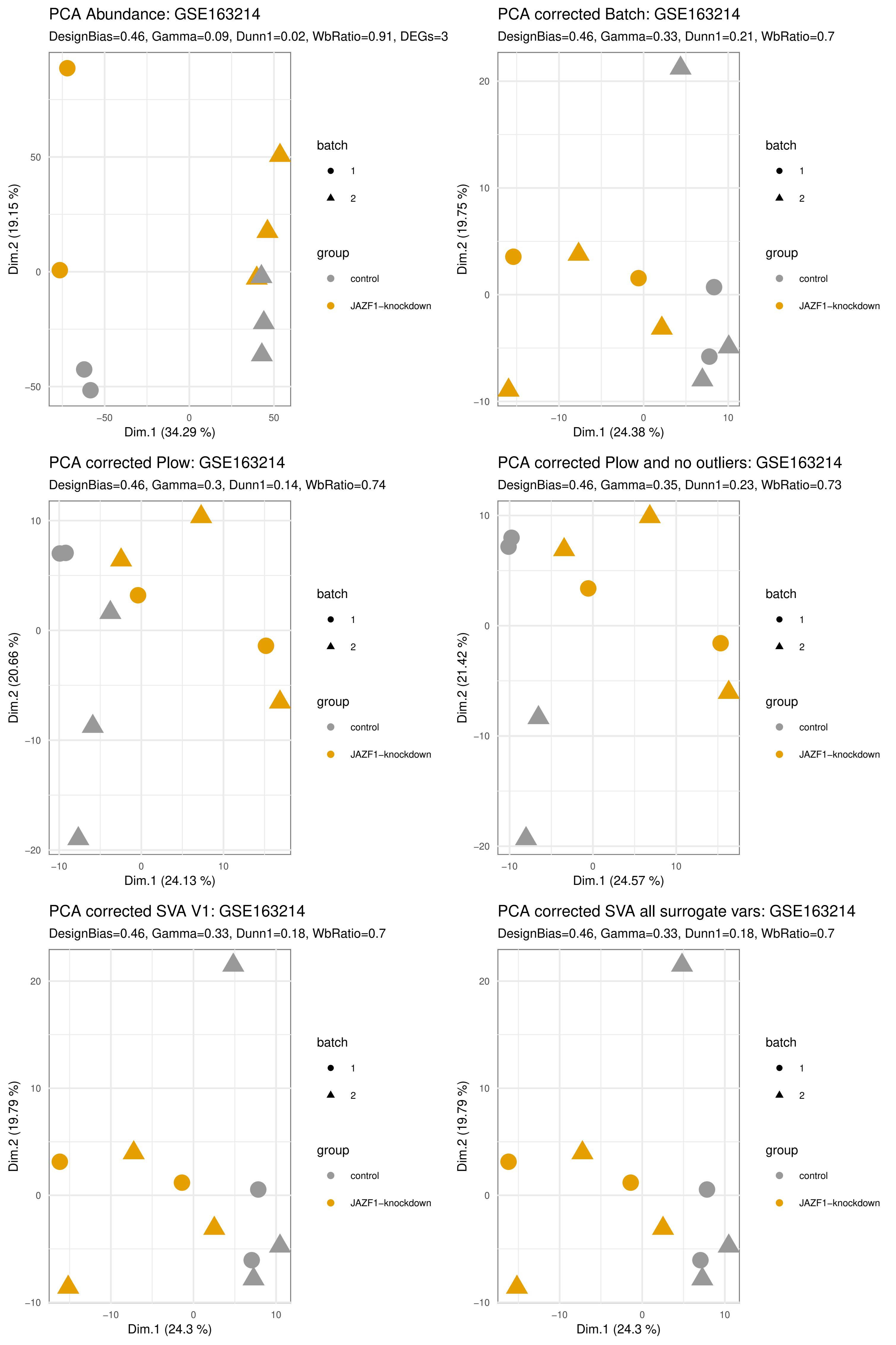
PCA Abundance: GSE163214
DesignBias=0.46, Gamma=0.09, Dunn1=0.02, WbRatio=0.91, DEGs=3
Dim.1 (34.29 %)
Dim.2 (19.15 %)
PCA corrected Batch: GSE163214
DesignBias=0.46, Gamma=0.33, Dunn1=0.21, WbRatio=0.7
Dim.1 (24.38 %)
Dim.2 (19.75 %)
PCA corrected Plow: GSE163214
DesignBias=0.46, Gamma=0.3, Dunn1=0.14, WbRatio=0.74
Dim.1 (24.13 %)
Dim.2 (20.66 %)
PCA corrected Plow and no outliers: GSE163214
DesignBias=0.46, Gamma=0.35, Dunn1=0.23, WbRatio=0.73
Dim.1 (24.57 %)
Dim.2 (21.42 %)
PCA corrected SVA V1: GSE163214
DesignBias=0.46, Gamma=0.33, Dunn1=0.18, WbRatio=0.7
Dim.1 (24.3 %)
Dim.2 (19.79 %)
PCA corrected SVA all surrogate vars: GSE163214
DesignBias=0.46, Gamma=0.33, Dunn1=0.18, WbRatio=0.7
Dim.1 (24.3 %)
Dim.2 (19.79 %)
batch
1
2
group
control
JAZF1−knockdown

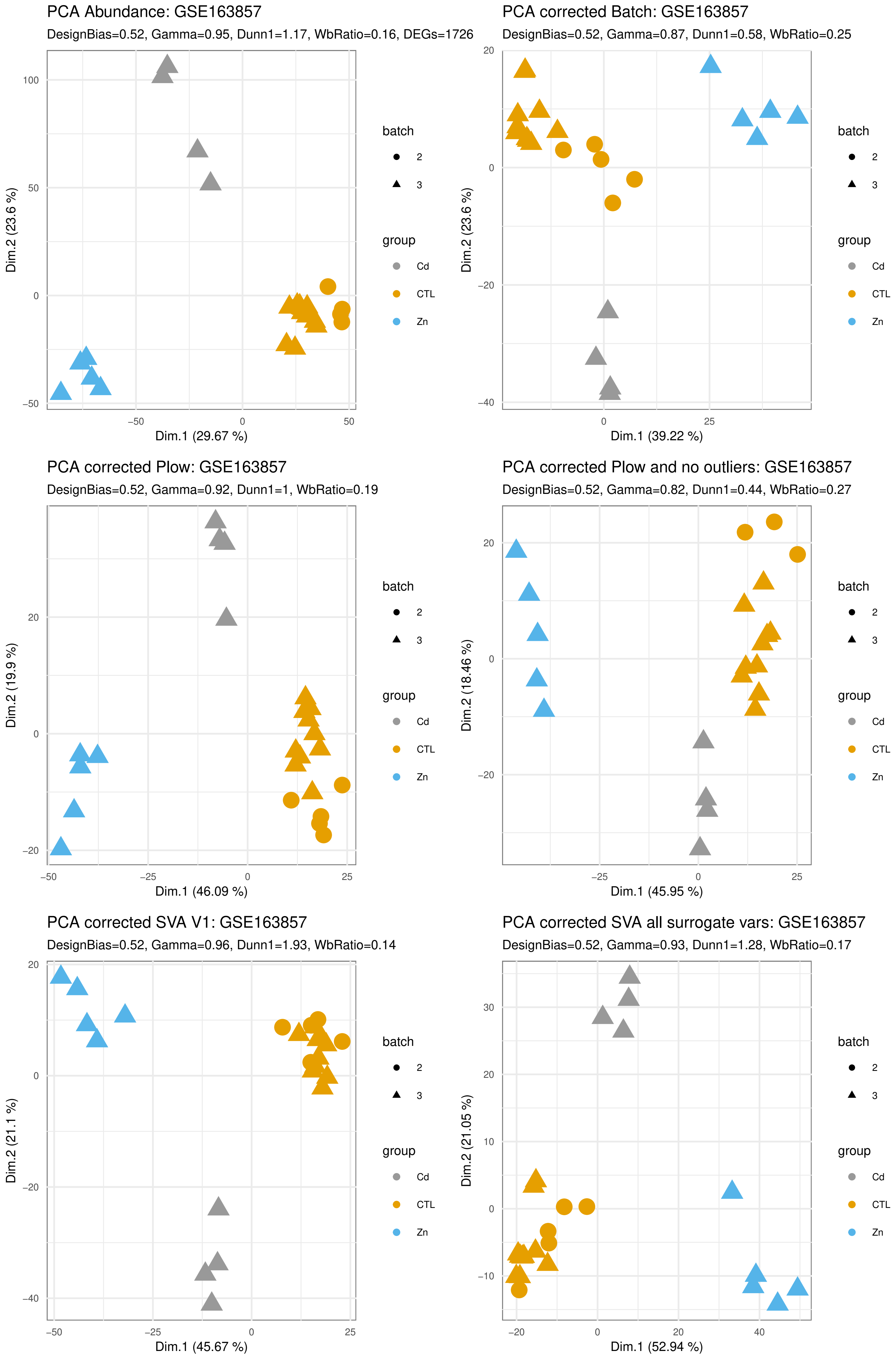
PCA Abundance: GSE163857
DesignBias=0.52, Gamma=0.95, Dunn1=1.17, WbRatio=0.16, DEGs=1726
Dim.1 (29.67 %)
Dim.2 (23.6 %)
PCA corrected Batch: GSE163857
DesignBias=0.52, Gamma=0.87, Dunn1=0.58, WbRatio=0.25
Dim.1 (39.22 %)
Dim.2 (23.6 %)
PCA corrected Plow: GSE163857
DesignBias=0.52, Gamma=0.92, Dunn1=1, WbRatio=0.19
Dim.1 (46.09 %)
Dim.2 (19.9 %)
PCA corrected Plow and no outliers: GSE163857
DesignBias=0.52, Gamma=0.82, Dunn1=0.44, WbRatio=0.27
Dim.1 (45.95 %)
Dim.2 (18.46 %)
PCA corrected SVA V1: GSE163857
DesignBias=0.52, Gamma=0.96, Dunn1=1.93, WbRatio=0.14
Dim.1 (45.67 %)
Dim.2 (21.1 %)
PCA corrected SVA all surrogate vars: GSE163857
DesignBias=0.52, Gamma=0.93, Dunn1=1.28, WbRatio=0.17
Dim.1 (52.94 %)
Dim.2 (21.05 %)
batch
2
3
group
Cd
CTL
Zn

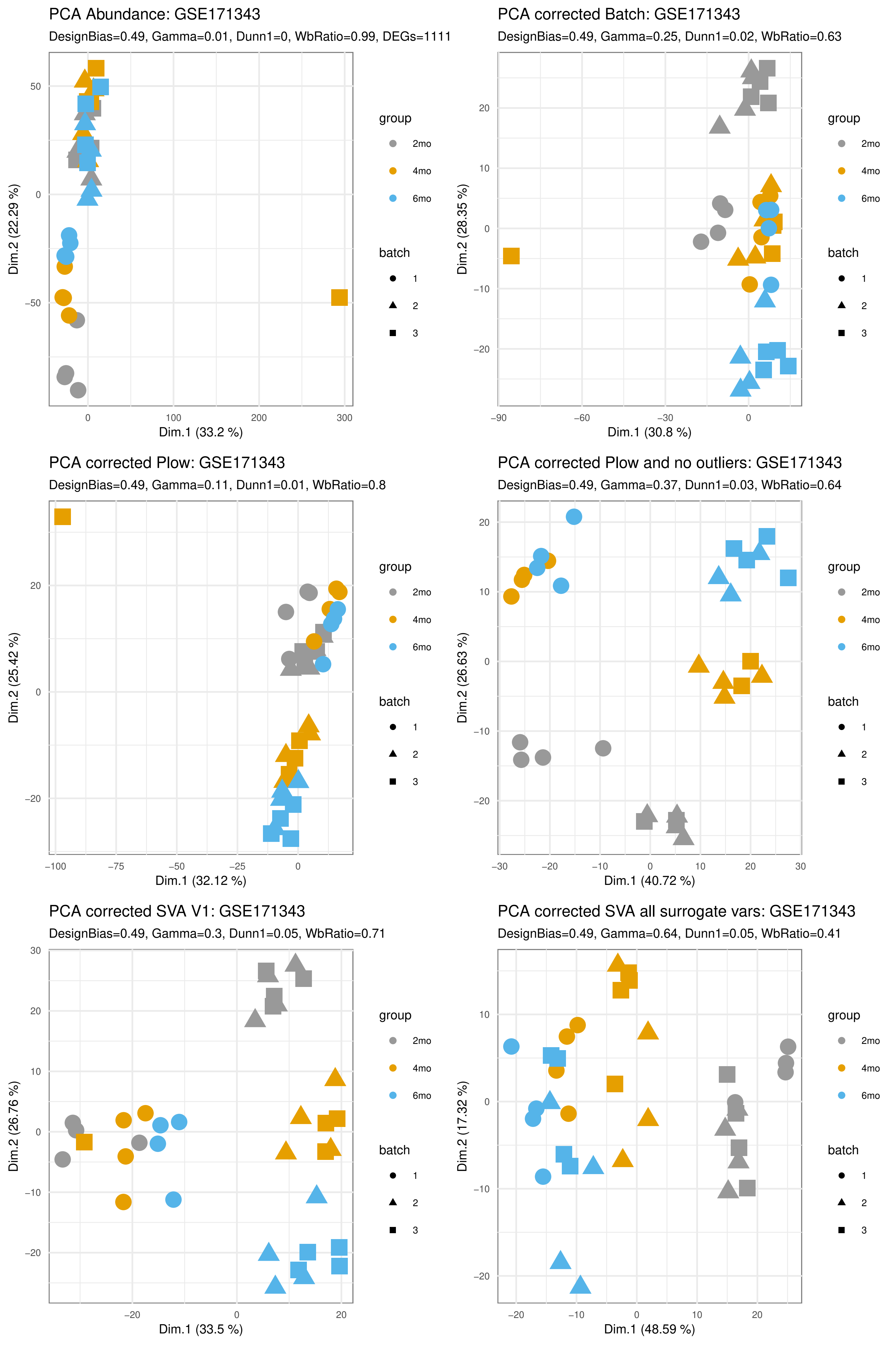
PCA Abundance: GSE171343
DesignBias=0.49, Gamma=0.01, Dunn1=0, WbRatio=0.99, DEGs=1111
Dim.1 (33.2 %)
Dim.2 (22.29 %)
PCA corrected Batch: GSE171343
DesignBias=0.49, Gamma=0.25, Dunn1=0.02, WbRatio=0.63
Dim.1 (30.8 %)
Dim.2 (28.35 %)
PCA corrected Plow: GSE171343
DesignBias=0.49, Gamma=0.11, Dunn1=0.01, WbRatio=0.8
Dim.1 (32.12 %)
Dim.2 (25.42 %)
PCA corrected Plow and no outliers: GSE171343
DesignBias=0.49, Gamma=0.37, Dunn1=0.03, WbRatio=0.64
Dim.1 (40.72 %)
Dim.2 (26.63 %)
PCA corrected SVA V1: GSE171343
DesignBias=0.49, Gamma=0.3, Dunn1=0.05, WbRatio=0.71
Dim.1 (33.5 %)
Dim.2 (26.76 %)
PCA corrected SVA all surrogate vars: GSE171343
DesignBias=0.49, Gamma=0.64, Dunn1=0.05, WbRatio=0.41
Dim.1 (48.59 %)
Dim.2 (17.32 %)
group
2mo
4mo
6mo
batch
1
2
3

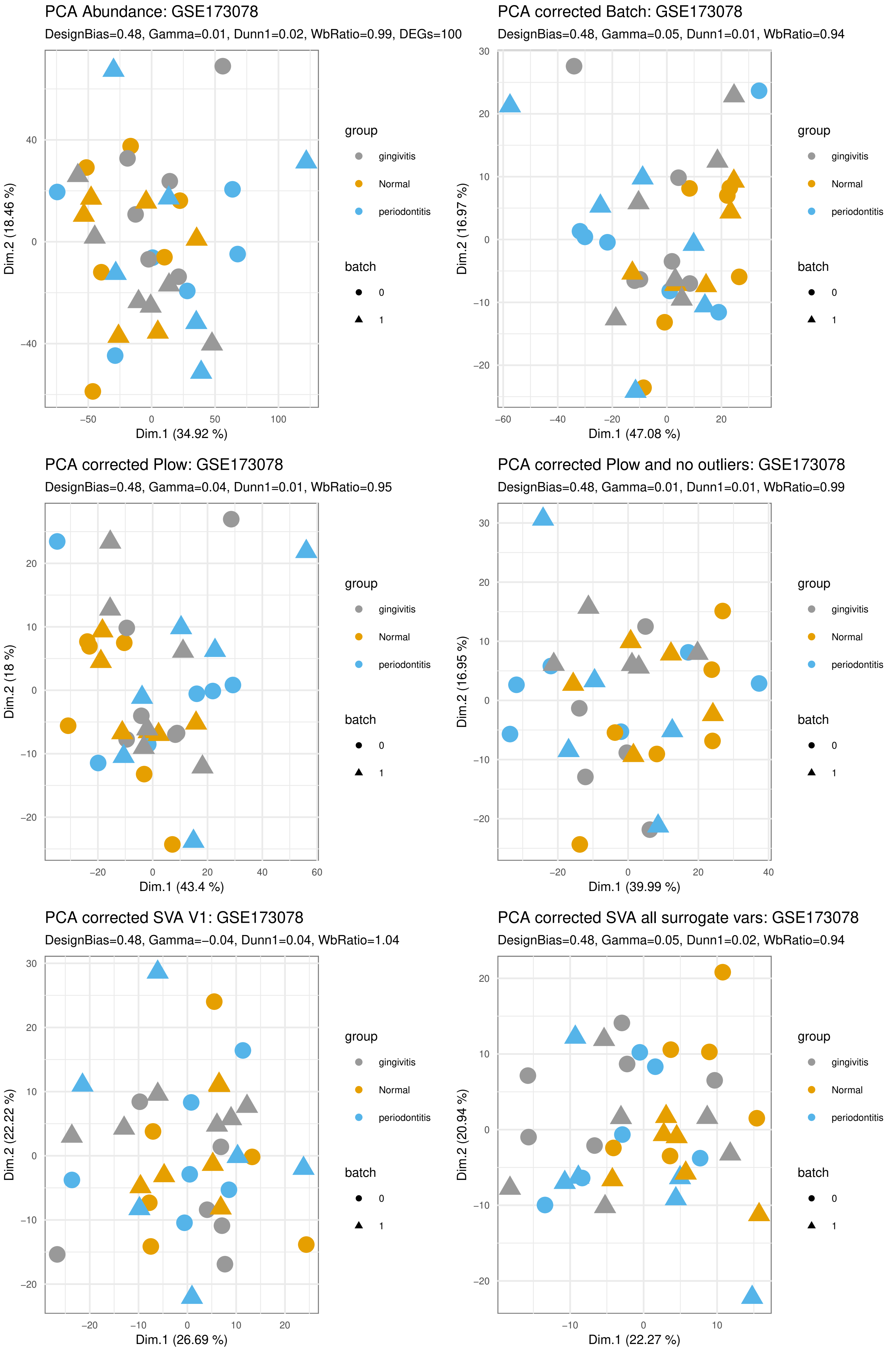
PCA Abundance: GSE173078
DesignBias=0.48, Gamma=0.01, Dunn1=0.02, WbRatio=0.99, DEGs=100
Dim.1 (34.92 %)
Dim.2 (18.46 %)
PCA corrected Batch: GSE173078
DesignBias=0.48, Gamma=0.05, Dunn1=0.01, WbRatio=0.94
Dim.1 (47.08 %)
Dim.2 (16.97 %)
PCA corrected Plow: GSE173078
DesignBias=0.48, Gamma=0.04, Dunn1=0.01, WbRatio=0.95
Dim.1 (43.4 %)
Dim.2 (18 %)
PCA corrected Plow and no outliers: GSE173078
DesignBias=0.48, Gamma=0.01, Dunn1=0.01, WbRatio=0.99
Dim.1 (39.99 %)
Dim.2 (16.95 %)
PCA corrected SVA V1: GSE173078
DesignBias=0.48, Gamma=−0.04, Dunn1=0.04, WbRatio=1.04
Dim.1 (26.69 %)
Dim.2 (22.22 %)
PCA corrected SVA all surrogate vars: GSE173078
DesignBias=0.48, Gamma=0.05, Dunn1=0.02, WbRatio=0.94
Dim.1 (22.27 %)
Dim.2 (20.94 %)
group
gingivitis
Normal
periodontitis
batch
0
1

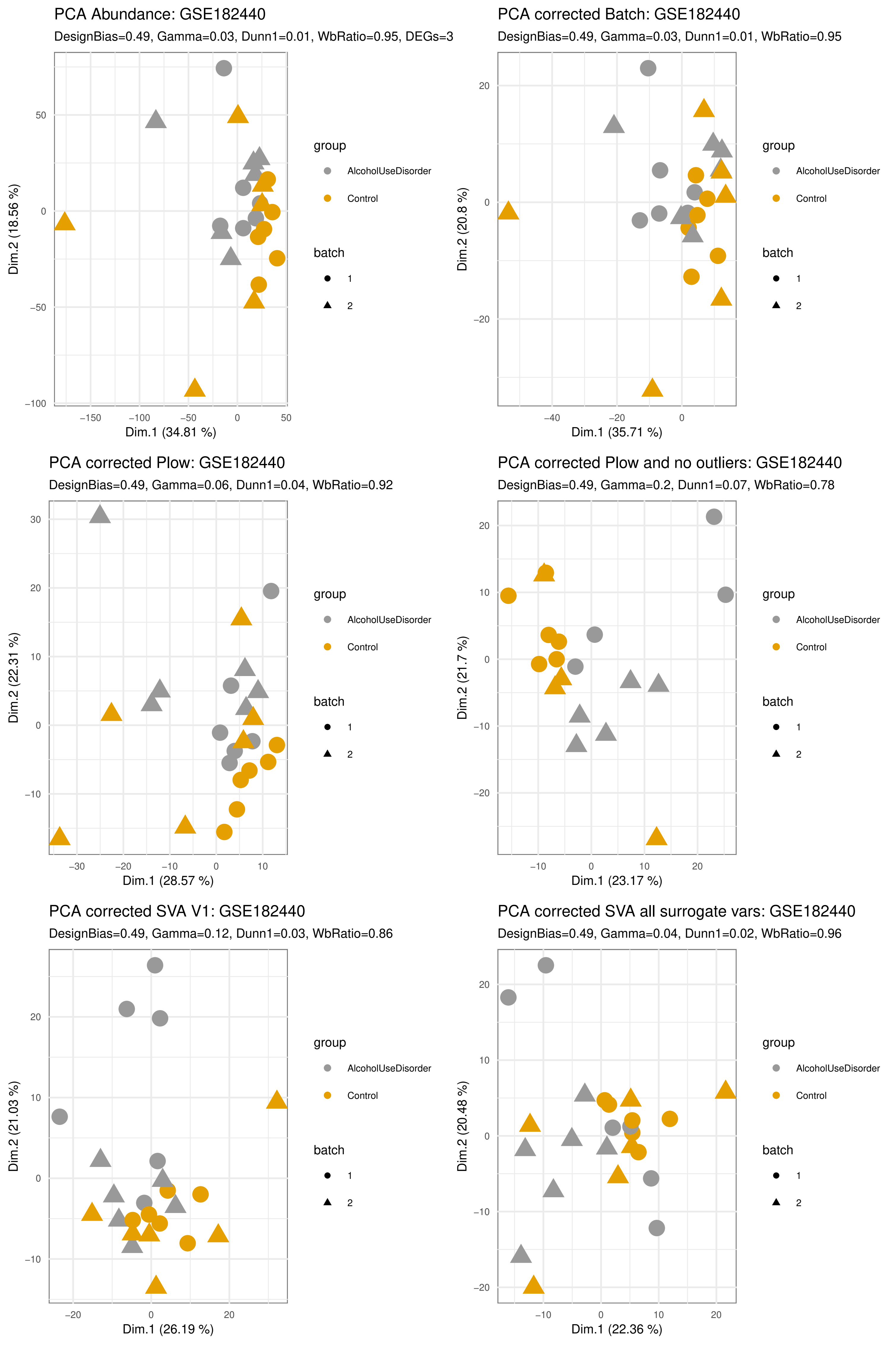

PCA Abundance: GSE182440
DesignBias=0.49, Gamma=0.03, Dunn1=0.01, WbRatio=0.95, DEGs=3
Dim.1 (34.81 %)
Dim.2 (18.56 %)
PCA corrected Batch: GSE182440
DesignBias=0.49, Gamma=0.03, Dunn1=0.01, WbRatio=0.95
Dim.1 (35.71 %)
Dim.2 (20.8 %)
PCA corrected Plow: GSE182440
DesignBias=0.49, Gamma=0.06, Dunn1=0.04, WbRatio=0.92
Dim.1 (28.57 %)
Dim.2 (22.31 %)
PCA corrected Plow and no outliers: GSE182440
DesignBias=0.49, Gamma=0.2, Dunn1=0.07, WbRatio=0.78
Dim.1 (23.17 %)
Dim.2 (21.7 %)
PCA corrected SVA V1: GSE182440
DesignBias=0.49, Gamma=0.12, Dunn1=0.03, WbRatio=0.86
Dim.1 (26.19 %)
Dim.2 (21.03 %)
PCA corrected SVA all surrogate vars: GSE182440
DesignBias=0.49, Gamma=0.04, Dunn1=0.02, WbRatio=0.96
Dim.1 (22.36 %)
Dim.2 (20.48 %)
group
AlcoholUseDisorder
Control
batch
1
2
